# Elevated temperature and carbon dioxide levels alter growth rates and shell composition in the fluted giant clam, *Tridacna squamosa*

**DOI:** 10.1038/s41598-022-14503-4

**Published:** 2022-06-30

**Authors:** Eric J. Armstrong, Sue-Ann Watson, Jonathon H. Stillman, Piero Calosi

**Affiliations:** 1 Department of Integrative Biology, University of California, 3040 Valley Life Sciences Building #3140, Berkeley, CA 94720-3140 USA; 2 Estuary & Ocean Science Center and Department of Biology, Romberg Tiburon Campus, San Francisco State University, 3150 Paradise Drive, Tiburon, CA 94920 USA; 3 Biodiversity and Geosciences Program, Museum of Tropical Queensland, Queensland Museum Network, Townsville, QLD 4810 Australia; 4 Australian Research Council Centre of Excellence for Coral Reef Studies, James Cook University, Townsville, QLD 4811 Australia; 5 Marine Biology & Ecology Research Center, University of Plymouth, Drake Circus, Plymouth, PL4 8AA UK; 6 Present Address: PSL Research University, EPHE, CNRS, Université de Perpignan, Perpignan, France; 7 Present Address: Département de Biologie, Chimie et Géographie, Université du Québec à Rimouski, 300 Allée Ursulines, Rimouski, QC G5L 3A1 Canada

**Keywords:** Marine biology, Ecophysiology, Animal physiology

## Abstract

Giant clams produce massive calcified shells with important biological (e.g., defensive) and ecological (e.g., habitat-forming) properties. Whereas elevated seawater temperature is known to alter giant clam shell structure, no study has examined the effects of a simultaneous increase in seawater temperature and partial pressure of carbon dioxide (*p*CO_2_) on shell mineralogical composition in these species. We investigated the effects of 60-days exposure to end-of-the-century projections for seawater temperature (+ 3 °C) and *p*CO_2_ (+ 500 µatm) on growth, mineralogy, and organic content of shells and scutes in juvenile *Tridacna squamosa* giant clams. Elevated temperature had no effect on growth rates or organic content, but did increase shell [^24^Mg]/[^40^Ca] as well as [^40^Ca] in newly-formed scutes. Elevated *p*CO_2_ increased shell growth and whole animal mass gain. In addition, we report the first evidence of an effect of elevated *p*CO_2_ on element/Ca ratios in giant clam shells, with significantly increased [^137^Ba]/[^40^Ca] in newly-formed shells. Simultaneous exposure to both drivers greatly increased inter-individual variation in mineral concentrations and resulted in reduced shell N-content which may signal the onset of physiological stress. Overall, our results indicate a greater influence of *p*CO_2_ on shell mineralogy in giant clams than previously recognized.

## Introduction

Giant clams (Tridacninae) inhabit nutrient-poor tropical coral reefs where they live in symbiosis with photosynthetic dinoflagellates of the family *Symbiodinaceae*. Within these biomes, giant clams are ecologically important species^1^ whose large shells provide habitat for numerous encrusting epibiota and which play a significant role in carbonate deposition/liberation^2^ and reef formation^1^. Because the strength and durability of clam shells depends on their elemental composition, significant research effort has been devoted to shedding light on the effects of environmental drivers on shell isotopic composition in giant clams^3–8^. In addition, as shell composition has the potential to provide useful information about conditions during biomineralization, there has been great interest in the influence of abiotic and biotic effects on incorporation of elemental impurities, especially Group II elements such as ^24^Mg, ^88^Sr, and ^137^Ba, which substitute for ^40^Ca within the calcium carbonate (CaCO_3_) lattice of giant clams’ shells^4,8–11^.

In contrast to scleractinian corals, for which environmental influences on carbonate element/Ca ratios are well-characterized(e.g., ^12^), our current understanding of the abiotic controls on trace element incorporation in *Tridacna* clams remains equivocal (see Table S1). Although shell [^24^Mg]/[^40^Ca], [^88^Sr]/[^40^Ca], and [^137^Ba]/[^40^Ca] ratios of giant clams appear strongly influenced by seawater temperature, and are even used as proxies for past sea surface temperature (SST)^4^, the exact nature of this influence, including its directionality, remains unclear^11^. In addition to this ambiguity regarding the influence of temperature, relatively few studies have examined the effects of multiple environmental drivers acting simultaneously on shell mineral structure in giant clams^13^, and none have investigated the effect of anthropogenically-driven increases in the partial pressure of carbon dioxide (*p*CO_2_) in seawater (i.e., ocean acidification) on element/Ca ratios in tridacnines. As alterations in shell mineral composition can have significant ecological impacts during a clam’s life (e.g., through reduced shell strength and thus increased vulnerability to crushing predators), as well as important repercussions for the durability and persistence of *Tridacna*-derived carbonate reefs, investigating mineralogical changes in response to multifaceted environmental change is an important first step for improving our understanding of current and projected global change impacts on giant clams and their associated reef habitats.

Knowledge of giant clam physiological responses to multiple climate-change stressors, including ocean warming and acidification, is vital for informing conservation strategies under rapid and ongoing environmental change^14^. Previous research has shown that exposure to elevated seawater temperature has profound impacts on giant clam physiology including decreased fertilization success^15^, reduced photosymbiont density^16,17^, altered lipid biosynthesis^18^, accumulation of reactive oxygen species^18^, altered holobiont oxygenic/respiratory balance^19,20^, and increased juvenile mortality^21^. Similarly, at elevated seawater *p*CO_2_, giant clams have been shown to grow more slowly^22^, build smaller shells^13,22,23^, and suffer higher mortality^21^ than under present-day ocean conditions^21,22^. Simultaneous prolonged (41 days) exposure to elevated *p*CO_2_ and elevated temperature reduced calcification rates and altered shell ultrastructure, increasing disordered crystalline lamellae^13^. Whereas these changes in shell crystalline structure suggest underlying alterations in shell mineral and organic content, no previous study has explicitly investigated paired mineralogical and organic responses in giant clam shells. In this study, we investigated the effects of elevated seawater temperature and *p*CO_2_ on the mineral composition and organic content of shells of the fluted giant clam *Tridacna squamosa* (Lamarck, 1819) to improve our understanding of physiological mechanisms related to reduced calcification in giant clams under projected future ocean conditions.

Because elevated seawater *p*CO_2_ results in decreased bioavailability of carbonate ions, we hypothesized that extended exposure of juvenile *T. squamosa* to elevated *p*CO_2_ would result in decreased shell growth rates and increased incorporation of trace mineral elemental impurities. Similarly, because elevated seawater temperature can result in holobiont stress (e.g., symbiotic breakdown and bleaching^24^) and alter trace element incorporation during biomineralization (Table S1), we hypothesized that exposure to elevated temperature would also decrease shell growth rates and alter mineralogical profiles in *T. squamosa* shells. Finally, we hypothesized that these two environmental drivers would interact synergistically when combined, leading to a greater reduction in shell growth and larger alterations of mineral profiles than when acting in isolation. To test these hypotheses, juvenile fluted giant clams were exposed to current and projected end-twenty-first century seawater conditions for 60 days, following which newly-formed (i.e., formed under treatment) and older-growth (i.e., formed prior to treatment) shell and scute were collected for analysis.

## Methods

### Specimen collection and exposure conditions

Juveniles of the giant clam *Tridacna squamosa* selected for this study (N = 32; shell length 35.81 ± 8.20 mm, mean ± s.d.) were spawned at the Darwin Aquaculture Centre (Wickham, Northern Territory, Australia) from wild-caught broodstock collected from the Northern Territory, Australia. Juvenile clams were transferred to the James Cook University aquarium facility where they were kept in natural seawater sourced from the Australian Institute of Marine Science seawater intake facility at Cape Cleveland until the experiment started. This natural seawater was filtered to 1 µm and UV-sterilized before introduction into the aquarium systems. Two > 8000 L recirculating seawater systems were maintained at two different partial pressures of carbon dioxide (*p*CO_2_) cross factored with two levels of temperature: + 0.0 and + 3.0 °C. Target values for seawater treatments were selected to mimic present-day and future, end-of-the-century, global ocean scenarios using the IPCC RCP 8.5 (business-as-usual) projections^25^ for temperature (28.5 and 30.5 °C, respectively^26,27^) and *p*CO_2_ (450 and 950 μatm, respectively^28,29^). Elevated CO_2_ treatments were achieved by dosing 100% CO_2_ into a 3000 L temperature-controlled sump on each system to a set pH using a pH control system (AT-Control, Aqua Medic, Germany) following standard techniques (Gattuso et al. 2010). Temperature (C22, Comark, Norwich, U.K.) and pH_NBS_ (HQ40d, Hach, Colorado, U.S.) were recorded daily in the treatment tanks. Realized temperature and CO_2_ levels for the four treatment conditions (2 × 2 design) were: (1) control temperature 28.7 °C and control *p*CO_2_ 436 µatm; (2) control temperature 28.8 °C and elevated *p*CO_2_ 929 µatm; (3) elevated temperature 31.3 °C and control *p*CO_2_ 449 µatm; and (4) elevated temperature 31.4 °C and elevated *p*CO_2_ 982 µatm. Salinity and total alkalinity were measured weekly. Total alkalinity was analyzed by Gran titration from water samples of replicate tanks in each system to within 1% of certified reference material (Prof. A.G. Dickson, Scripps Institution of Oceanography). Seawater *p*CO_2_ was calculated using the program CO2SYS^30^ with the constants of Mehrbach et al*.*^31^ refit by Dickson and Millero^32^ and Dickson^33^ for K(HSO_4_^−^). Water conditions for each treatment are reported in Table 1. Light levels, measured as photosynthetically active radiation (PAR), were 340 µmol photons m^−2^ s^−1^. Clams were randomly assigned to one of the four treatment conditions (N = 8 clams *per* treatment) where they were held for 60 days before samples of both newly-formed and older-growth shell and scute were collected.

**Table 1:** Seawater carbonate chemistry for experimental treatments.

Treatment	Temp (°C)	Salinity	*p*CO_2_ (µatm)	pH_NBS_	Total alkalinity (µmol kg^−1^ SW)	Ω_Ca_	Ω_Ar_
Ambient control	28.7 ± 0.0	35.0 ± 0.2	435.5 ± 7.9	8.14 ± 0.01	2201.9 ± 5.7	5.03 ± 0.07	3.35 ± 0.04
Elevated *p*CO_2_	28.8 ± 0.0	34.9 ± 0.1	929.1 ± 14.5	7.86 ± 0.01	2183.7 ± 10.1	2.94 ± 0.04	1.96 ± 0.02
Elevated temp	31.3 ± 0.1	35.0 ± 0.2	448.6 ± 9.2	8.14 ± 0.01	2201.9 ± 5.7	5.31 ± 0.07	3.57 ± 0.05
Multistressor	31.4 ± 0.0	34.9 ± 0.1	982.2 ± 16.9	7.85 ± 0.01	2183.7 ± 10.1	3.07 ± 0.04	2.07 ± 0.02

Values are means ± s.e. to nearest integer, 1 or 2 d.p. as appropriate.

### Shell morphometry

**Figure 1:**
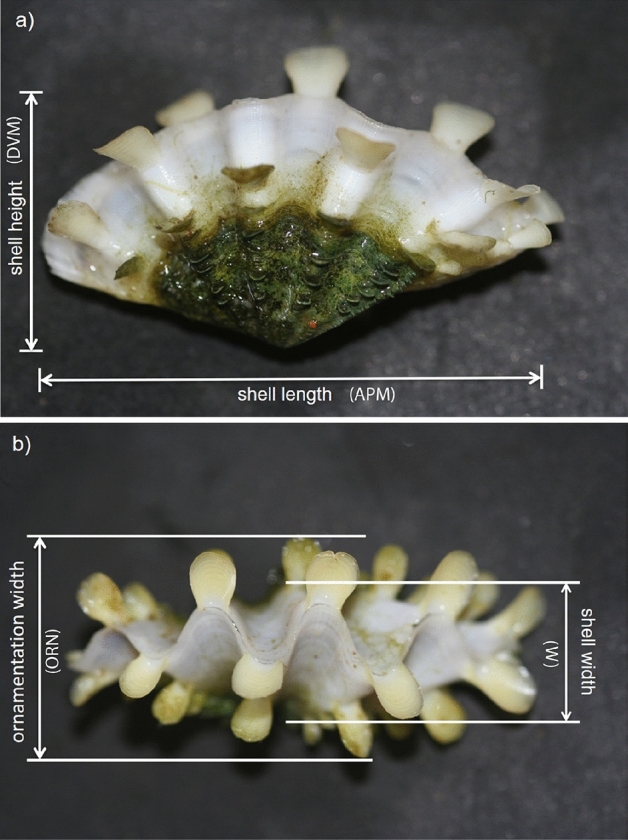
Morphometric measurements of the shell ((**a**) lateral view; (**b**) dorsal view) of the fluted giant clam *Tridacna squamosa*: anterior–posterior margin (APM; shell length); dorsal–ventral margin (DVM; shell height); shell width (W); and maximum width as measured across projecting scutes (ORN; ornamentation width).

The following five morphological parameters were measured using digital calipers on *T. squamosa* shells both pre- and post-exposure to experimental conditions: shell anterior–posterior margin (i.e., shell length, Fig. 1a), shell dorsal–ventral margin (i.e., shell height, Fig. 1a), shell width (Fig. 1b), shell width as measured across scutes (i.e. ornamentation width, Fig. 1b), and total wet weight of the intact clam (Table S2). Shell growth over the 60-days exposure period was calculated for each clam as the proportional difference between morphometric variables at the start and end of the exposure period (i.e., % change).

### Shell mineralogy

For mineralogical analyses, fragments of various growth regions (i.e., new-growth from the dorsal margin and older-growth from near the ventral margin) of *T. squamosa* shell and scute were crushed into a fine powder using a clean, porcelain, pestle and mortar, which was thoroughly rinsed with MilliQ ultrapure water and blotted dry with filter paper in between individual shell preparations to avoid cross-contamination. Approximately 0.5–1.5 mg of each powdered shell sample was then completely dissolved in 10 mL 2% HNO_3_ (75% TraceMetalTM Grade, Fisher Chemical, Loughborough, UK) in a microwave reaction system (MARS 6, CEM Corporation, Matthews, NC, USA). Ionic concentrations (i.e., ^40^Ca, ^24^Mg, ^28^Si, ^31^P, ^39^K, ^55^Mn, ^75^As, ^88^Sr, and ^137^Ba) were measured from aliquots of this solution using inductively-coupled-plasma mass spectrometry (ICP-MS, X-Series II, Thermo Scientific, Hemel Hempstead, UK) following the method of Wolf and Adams (2015)^34^. When necessary (e.g., for ^40^Ca), 200-fold dilutions of prepared samples were made in 2% HNO_3_. Samples were run in triplicate and all elements were analyzed simultaneously. Raw elemental concentration data for ^40^Ca and ^88^Sr were drift-corrected using the sample-standard correction method described by Schrag^35^.

### Shell organic content

Elemental analysis was performed on subsamples of powdered shell (mass range: 1.51–3.31 mg) after combustion in a CHN Elemental Analyser (Model EA1110, CE Instruments, Wigan, UK). Because preliminary analyses of bivalve shell material revealed a carbon content of ca. 12% by mass, l-cysteine (ca. 30% carbon by mass) was used as a standard for calibration of all *T. squamosa* shell samples. Samples were run in duplicate (data reported as means) and all elements (i.e., C, H, and N) were analyzed simultaneously.

### Statistical analyses

The effects of elevated seawater temperature and *p*CO_2_ and their interaction on morphometric components of new shell growth (i.e., clam wet mass gain, shell length gain, shell height gain, shell width gain, and ornamentation width gain) were analyzed using a two-way multivariate analysis of covariance (*MANCOVA*), with temperature and *p*CO_2_ as fixed factors. As all pre-exposure morphometric variables were strongly, and significantly correlated (Table S2), we used only one of them (i.e., pre-exposure shell length) as a covariate in this initial *MANCOVA*. As a follow-up to the *MANCOVA*, a series of two-way *ANCOVA*s were conducted on each of the five morphometric response variables using the corresponding pre-exposure morphometric trait as the covariate: e.g. pre-exposure shell length for length gain analysis, etc.

Unlike morphometric response variables, which were only quantified once *per* individual at the end of the experiment (i.e., growth over the course of treatment), mineralogical and organic data were collected multiple times for each individual (i.e., sampling of both newly-formed and older-growth shell and scute). For analysis of these data, any measurement with a value less than zero (i.e., a negative elemental concentration indicating a value below the respective limit of detection for that ion) was assigned a value of zero. In addition, measurements with values greater than 10x (mineralogical data) or 5x (organic content data) their respective group mean were removed from analyses as probable technical outliers (N = 18 and N = 1 values removed, respectively). After outlier removal, a total of N = 966 mineralogical and N = 77 organic content values were retained for further analysis.

We then used a series of paired t-tests to determine if there were significant differences in any of the response variables between shell and scute. Because differences were observed between newly-formed shell and newly-formed scute, all mineralogical and organic variables were analyzed separately for shell and scute in subsequent analyses. As explained previously for morphometric data, a similar suite of *MANCOVA*s (and follow-up *ANCOVA*s) were run on the shell organic and mineral content data using post-exposure total wet mass as the covariate.

Before performing *MANCOVA*s for morphometric, organic, and mineralogical variables, assumptions of normality were visually assessed for all response variables (i.e., five morphometric variables, three organic variables, and nine mineralogical variables) with Q-Q plots. Homogeneity of variance was tested for all variables using Levene’s *F* test. Based on Levene’s *F* test results, the homogeneity of variance assumption was satisfied for all organic variables but was not satisfied for one of the five morphological variables and several of the mineralogical variables (i.e., *P* < 0.05, see Table S3). Specifically, although the Levene’s *F* tests suggested that the variances associated with the shell width gain morphological variable were not homogenous, an examination of the variances revealed that none of the largest variances were more than four times the size of the corresponding smallest, suggesting that the *ANCOVA* would be robust to non-homogenous variance in this case^36^. However, for the mineralogical variables, the largest variances were greater than four times the smallest in all cases. Thus, to permit further parametric hypothesis testing, all mineralogical data, except for element/Ca ratios, for both scute and shell were transformed (see below) to satisfy homogeneity of variance assumptions.

For transformation of the mineralogical data, optimization of the Box-Cox parameter lambda (λ) indicated that square-root transformation (λ = 0.5) and log_10_ transformation (λ = 0) were roughly equivalent. Thus, we chose to square-root transform all mineralogical variables to avoid complications arising from mineral concentrations equal to zero. Subsequent Levene’s *F* tests analyzing homogeneity of variance on square-root-transformed mineralogical data were non-significant for all minerals (all *P* > 0.05) save for [^31^P] and [^39^K] in newly-formed shell (F_3, 27_ = 3.49, *P* = 0.03, and F_3, 27_ = 4.29, *P* = 0.01, respectively). However, as log-transformation did not improve Levene’s *F* test outcomes for these three variables, square-root-transformed data were used in follow-up parametric models.

For all *ANCOVA*s, covariates and interactions between the fixed factor and covariates were dropped when not significant. All ANCOVAs were performed using the *aov* and *Anova* functions from the “stats” (v. 4.0.2) and “car” (v. 3.0.9) packages, respectively^37^, in the statistical computing software program R (v. 3.6.1)^38^, and results were considered statistically significant (moderate evidence of an effect) and marginally significant (weak evidence of an effect) at alpha values ≤ 0.05 and between 0.05 and 0.1, respectively. Finally, a series of post-hoc analyses (i.e. Bonferroni pairwise comparisons) were performed using estimated marginal means calculated with the R function *emmeans* in the package “emmeans” (v. 1.5.0)^39^ with FDR (i.e. Bonferroni) multiple-comparison p-adjustments to examine individual mean difference comparisons across all levels of experimental treatment. The effect sizes (i.e., partial η^2^) were calculated using the *etaSquared* function of the “lsr” package (v. 0.5)^40^ and are reported for significant comparisons in Table 2 (shell morphometrics and organic content), Table 3 (mineral content of newly-formed scute/shell), and Table 4 (mineral content of older-growth scute/shell). A summary of all ANCOVA results (including non-significant tests) are provided for morphometrics and organic content (Table S4), mineralogical content in newly-formed scute and shell (Tables S5, S6, respectively) and in older-growth scute and shell (Tables S7, S8, respectively).

**Table 2:** Results of significant (*) and marginally significant (˟) 2-way *ANCOVA*s and pairwise comparisons (EMMs) examining the effect of elevated seawater temperature and *p*CO_2_ on scute and shell morphometric traits and organic content of newly-formed shell in the fluted giant clam *Tridacna squamosa*.

Trait	ANCOVA				EMMs		Summary statistics			
	Factor	F_(1, 27)_	*P*	*η*_*p*_^*2*^	Pairwise contrast	*P*	Treatment	*N*	M	SD
Total wet mass gain (% change)	*p*CO_2_	6.03	0.02*	0.18	Ambient vs elevated *p*CO_2_	0.06^×^	Ambient	8	54.43	43.93
							Elevated pCO_2_	8	90.36	40.34
					Elevated temp vs elevated *p*CO_2_	0.04*	Elevated temp	8	46.43	23.70
							Multistressor	8	70.80	33.62
	Initial total wet mass (cov)	3.55	0.07^×^	0.12	–	–				
Shell length gain (% change)	*p*CO_2_	9.43	< 0.01*	0.26	Ambient vs elevated pCO_2_	0.03*	Ambient	8	9.12	8.34
					Ambient vs multistressor	0.07^×^	Elevated *p*CO_2_	8	22.83	9.05
					Elevated temp vs elevated *p*CO_2_	0.07^×^	Elevated temp	8	13.04	9.55
							Multistressor	8	19.96	7.25
Shell width gain (% change)	*p*CO_2_	8.01	0.01*	0.23	Ambient vs elevated pCO_2_	0.02*	Ambient	8	14.96	13.30
							Elevated *p*CO_2_	8	25.94	9.27
					Elevated temp vs elevated *p*CO_2_	< 0.01*	Elevated temp	8	12.04	5.29
					Elevated temp vs multistressor	0.04*	Multistressor	8	20.49	7.33
	Initial shell width (cov)	3.77	0.06^×^	0.12	*–*	–				
Shell height gain (% change)	*p*CO_2_	4.85	0.04*	0.15	Ambient vs elevated *p*CO_2_	0.07^×^	Ambient	8	16.53	12.14
							Elevated *p*CO_2_	8	25.95	11.60
					Elevated temp vs elevated *p*CO_2_	0.06^×^	Elevated temp	8	14.09	7.53
							Multistressor	8	23.12	9.82
					Elevated temp vs multistressor	0.07^×^				
Ornamentation width gain (% change)	*p*CO_2_	5.66	0.02*	0.07	Ambient vs elevated *p*CO_2_	0.05*	Ambient	8	20.52	19.27
					Ambient vs multistressor	0.05*	Elevated *p*CO_2_	8	37.02	16.65
							Elevated temp	8	19.70	11.16
					Elevated temp vs elevated *p*CO_2_	0.05*	Multistressor	8	34.74	15.11
					Elevated temp vs multistressor	0.05*				
Nitrogen content (weight %)	Temperature	F_(1, 20)_ 3.10	0.09^×^	0.13	No significant pairwise contrasts	–	Ambient	6	0.09	0.12
							Elevated *p*CO_2_	7	0.03	0.04
							Elevated temp	7	0.03	0.03
							Multistressor	5	0.00	0.00

Factors with significant (*) or marginal effects (˟) on investigated traits are noted. *ANCOVA* results for minerals are based on square-root transformed data whereas summary statistics are for non-transformed data.

*Trait* response variable, *Factor* independent variable including fixed factors and covariate (cov), *F* F-value, *P* p-value, *η*_*p*_^*2*^ partial eta squared, *N* number of samples, *M* group mean (% change, concentration in mmol kg^−1^, or ratio), and *SD* group standard deviation.

**Table 3:** Results of significant (*) and marginally significant (˟) 2-way *ANCOVA*s and pairwise comparisons (EMMs) examining the effect of elevated seawater temperature and *p*CO_2_ on mineral content of newly-formed scute and shell in the fluted giant clam *Tridacna squamosa*.

Trait	ANCOVA				EMMs		Summary statistics			
	Factor	F_(1, 26)_	P	η_p_^2^	Pairwise Contrast	P	Treatment	N	M	SD
New scute [Calcium] (mmol kg^−1^)	Temperature	F_(1, 25)_ 8.05	0.01*	0.24	Ambient vs elevated temp	0.05*	Ambient	6	4700.37	3111.39
							Elevated *p*CO_2_	8	8636.72	5014.59
	Interaction	3.04	0.09^×^	0.11	No significant pairwise contrasts	–	Elevated temp	8	19,178.69	21,800.54
							Multistressor	8	10,122.93	9164.46
	Post-exposure wet mass (cov)	10.67	< 0.01*	0.30	–	–				
New scute [Barium] (mmol kg^−1^)	*p*CO_2_	F_(1, 25)_ 4.20	0.05*	0.14	No significant pairwise contrasts	–	Ambient	7	0.01	0.01
							Elevated *p*CO_2_	8	0.01	0.01
							Elevated temp	7	0.01	0.02
	Post-exposure wet mass (cov)	8.26	0.01*	0.25	–	–	Multistressor	8	0.02	0.02
New shell [Barium] (mmol kg^−1^)	*p*CO_2_	4.62	0.04*	0.15	Ambient vs elevated pCO_2_	0.08^×^	Ambient	8	0.00	0.01
							Elevated *p*CO_2_	8	0.03	0.05
					Ambient vs multistressor	0.01*	Elevated temp	8	0.01	0.01
					Elevated temp vs multistressor	0.01*	Multistressor	7	0.06	0.05
New shell [Potassium] (mmol kg^−1^)	Interaction	3.79	0.06^×^	0.13	No significant pairwise contrasts	–	Ambient	8	4.35	3.73
							Elevated *p*CO_2_	8	4.53	7.51
							Elevated temp	8	1.78	0.80
							Multistressor	7	11.69	11.15
New shell [^24^Mg]/[^40^Ca] (mmol/mol)	Temperature	7.38	0.01*	0.22	Ambient vs elevated temp	0.07^×^	Ambient	8	0.49	0.14
							Elevated *p*CO_2_	8	0.60	0.09
							Elevated temp	8	0.65	0.08
							Multistressor	7	0.66	0.14
	Post-exposure wet mass (cov)	3.23	0.08^×^	0.11	–	–				
New shell [^137^Ba]/[^40^Ca] (µmol/mol)	*p*CO_2_	8.98	< 0.01*	0.26	Ambient vs elevated pCO_2_	0.04*	Ambient	8	0.27	0.44
							Elevated *p*CO_2_	8	1.17	0.46
							Elevated temp	8	0.55	0.76
							Multistressor	7	0.82	0.71

Factors with significant (*) or marginal effects (˟) on investigated traits are noted. *ANCOVA* results for minerals are based on square-root transformed data whereas summary statistics are for non-transformed data.

*Trait* response variable, *Factor* independent variable including fixed factors and covariate (cov), *F* F-value, *P* p-value, *η*_*p*_^*2*^ partial eta squared, *N* number of samples, *M* group mean (% change, concentration in mmol kg^−1^, or ratio), and *SD* group standard deviation.

**Table 4:** Results of significant (*) and marginally significant (˟) 2-way *ANCOVA*s and pairwise comparisons (EMMs) examining the effect of elevated seawater temperature and *p*CO_2_ on mineral content of older-growth scute and shell in the fluted giant clam *Tridacna squamosa*.

Trait	ANCOVA				EMMs		Summary statistics			
	Factor	F_(1, 26)_	*P*	*η*_*p*_^*2*^	Pairwise contrast	*P*	Treatment	*N*	M	SD
Old scute [Strontium] (mmol kg^−1^)	*p*CO_2_	F_(1, 23)_ 3.65	0.07˟	0.14	No significant pairwise contrasts	–	Ambient	7	5.29	8.59
	Post-exposure wet mass (cov)	3.84	0.06˟	0.02	–	–	Elevated *p*CO_2_	8	26.21	38.68
							Elevated temp	7	12.63	10.24
							Multistressor	6	26.86	39.24
Old scute [Barium] (mmol kg^−1^)	*p*CO_2_	F_(1, 24)_ 5.00	0.03*	0.17	No significant pairwise contrasts	–	Ambient	7	0.01	0.01
	Post-exposure wet mass (cov)	9.08	0.01*	0.27	–	–	Elevated *p*CO_2_	8	0.02	0.03
							Elevated temp	7	0.01	0.01
							Multistressor	7	0.03	0.04
Old shell [Calcium] (mmol kg^−1^)	Temperature	3.91	0.06^×^	0.13	No significant pairwise contrasts	–	Ambient	7	109,510.05	156,705.07
	Interaction	4.67	0.04*	0.15	No significant pairwise contrasts	–	Elevated *p*CO_2_	8	35,689.52	40,808.94
							Elevated temp	8	12,553.36	6801.87
							Multistressor	8	80,249.66	126,975.81
Old shell [Magnesium] (mmol kg^−1^)	Temperature	6.18	0.02*	0.19	Ambient vs ELEVATED Temp	0.09^×^	Ambient	7	0.17	0.25
	Interaction	5.72	0.02*	0.18	No significant pairwise contrasts	–	Elevated *p*CO_2_	8	0.02	0.02
							Elevated temp	8	0.00	0.00
							Multistressor	8	0.08	0.10
Old shell [Manganese] (mmol kg^−1^)	*p*CO_2_	4.61	0.04*	0.15	No significant pairwise contrasts	–	Ambient	7	0.17	0.25
	Temperature	7.75	0.01*	0.23	Ambient vs elevated temp	0.06^×^				
	Interaction	6.98	0.01*	0.21	No significant pairwise contrasts	–	Elevated *p*CO_2_	8	0.02	0.02
							Elevated temp	8	0.00	0.00
							Multistressor	8	0.08	0.10
Old shell [Barium] (mmol kg^−1^)	Interaction	3.70	0.07^×^	0.12	No significant pairwise contrasts	–	Ambient	7	0.25	0.36
							Elevated *p*CO_2_	8	0.09	0.13
							Elevated temp	8	0.03	0.01
							Multistressor	8	0.23	0.44
Old shell [Silicon] (mmol kg^−1^)	Temperature	6.23	0.02*	0.19	No significant pairwise contrasts	–	Ambient	7	58.14	77.77
	Interaction	4.81	0.04*	0.16	No significant pairwise contrasts	–				
							Elevated *p*CO_2_	8	15.02	14.48
							Elevated temp	8	5.26	5.24
							Multistressor	8	28.72	31.21
Old shell [Phosphorus^−^] (mmol kg^−1^)	Temperature	5.54	0.03*	0.18	No significant pairwise contrasts	–	Ambient	7	0.82	0.27
	Interaction	5.10	0.03*	0.16	No significant pairwise contrasts	–	Elevated *p*CO_2_	8	0.75	0.30
							Elevated temp	8	0.59	0.25
							Multistressor	8	0.56	0.23
Old shell [Potassium] (mmol kg^−1^)	Temperature	6.22	0.02*	0.19	No significant pairwise contrasts	–	Ambient	7	1.73	0.53
	Interaction	5.27	0.03*	0.17	No significant pairwise contrasts	–	Elevated *p*CO_2_	8	2.97	1.45
							Elevated temp	8	2.90	1.42
							Multistressor	8	2.87	1.81
Old shell [^24^Mg]/[^40^Ca] (mmol/mol)	Temperature	5.16	0.03*	0.16	Ambient vs multistressor	0.03*	Ambient	7	2.38	0.42
	Post-exposure Wet Mass (cov)	8.66	0.01*	0.24	–	–	Elevated pCO_2_	8	2.51	0.98
							Elevated temp	8	2.35	1.02
							Multistressor	8	2.17	0.95

Factors with significant (*) or marginal effects (˟) on investigated traits are noted. *ANCOVA* results for minerals are based on square-root transformed data whereas summary statistics are for non-transformed data.

*Trait* response variable, *Factor* independent variable including fixed factors and covariate (cov), *F* F-value, *P* p-value, *η*_*p*_^*2*^ partial eta squared, *N* number of samples, *M* group mean (% change, concentration in mmol kg^−1^, or ratio), and *SD* group standard deviation.

## Results

### Shell morphometry

We observed a marginal effect of *p*CO_2_ on aggregate shell morphometry (MANCOVA, F_5, 23_ = 2.53, *P* = 0.06, Pillai’s Trace = 0.35) with increased shell growth rates under elevated *p*CO_2_. The multivariate effect size (partial η^2^ or Pillai’s score) of *p*CO_2_ on overall change in shell morphology indicated that 35% of the variance was accounted for by elevated seawater *p*CO_2_ during shell formation. Subsequent ANCOVA analyses for each of the five morphometric response variables revealed a statistically significant effect of *p*CO_2_ on shell growth rates in that variable (Table 2). Effect sizes (partial η^2^) of *p*CO_2_ ranged from a low of 0.15 (shell height gain) to a high of 0.26 (shell length gain; Table S4).

**Figure 2:**
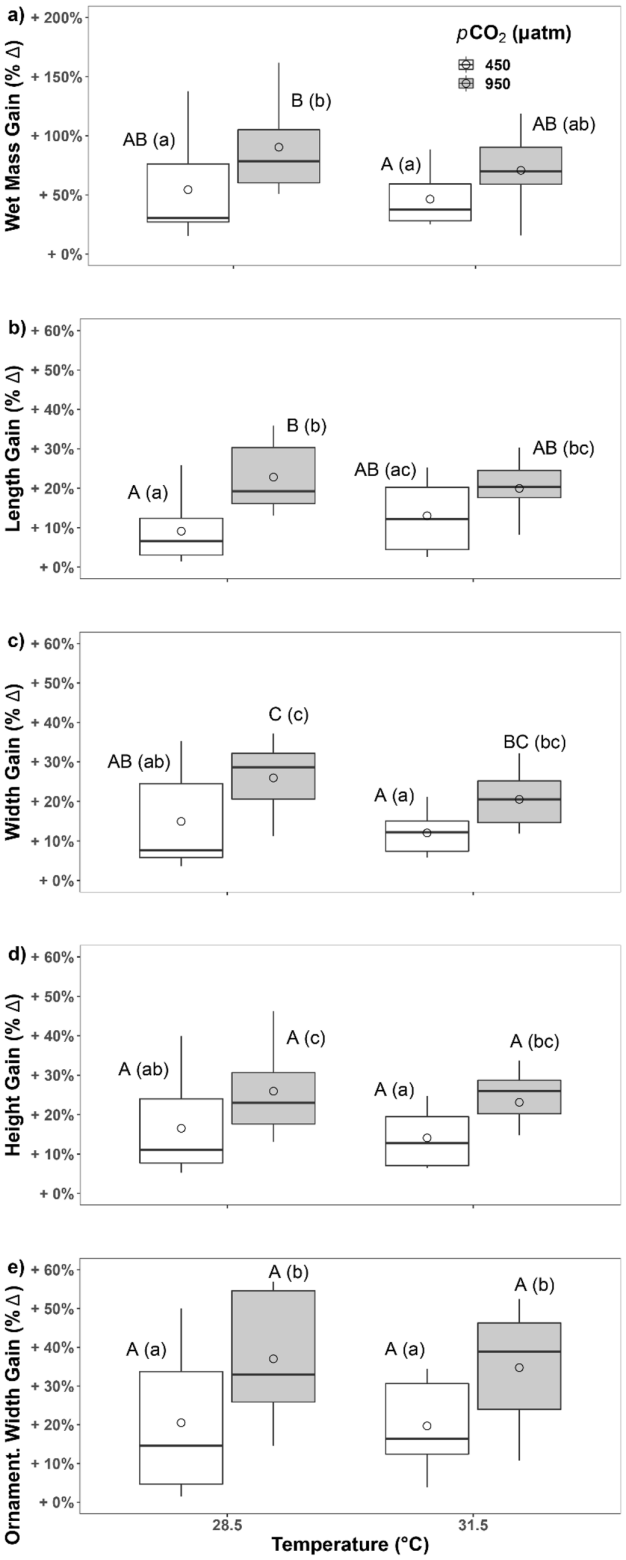
The effects of 60-days exposure to elevated temperature (28.5 and 31.5 ℃) and *p*CO_2_ (450 and 950 µatm) on percentage change in *T. squamosa* shell morphometric traits: (**a**) whole individual wet mass, (**b**) shell length, (**c**) shell width, (**d**) shell height, and (**e**) ornamentation width (scutes). Boxplots display group means (dots), medians (horizontal dark bar), and interquartile (upper and lower box horizontal lines) and 1.5× interquartile ranges (whiskers). White boxes depict traits measured at 450 µatm *p*CO_2_ and grey at 950 µatm *p*CO_2_. Significantly (P ≤ 0.05) and marginally (0.05 < P ≤ 0.1) different means, according to estimated marginal means (EMMs) tests with FDR-Bonferroni correction, are indicated by different upper- and lowercase letters, respectively.

Examining each variable individually, we observed that total clam wet mass gain (% change) differed among treatments (ANCOVA, F_1, 27_ = 6.03, *P* = 0.02; Table 2), with significantly greater mass gain at elevated *p*CO_2_ than at elevated temperature (Bonferroni EMMs post-hoc test, t-ratio_1, 27_ =  − 2.93, *P* = 0.04; Fig. 2a). Total wet mass gain was also marginally higher at elevated *p*CO_2_ than at ambient conditions (Bonferroni EMMs post-hoc test, t-ratio_1, 27_ =  − 2.46, *P* = 0.06). Shell length gain (% change) also differed between treatments (ANCOVA, F_1, 27_ = 9.43, *P* < 0.01; Table 2), with significantly greater length gain at elevated *p*CO_2_ than at ambient conditions (Bonferroni EMMs post-hoc test, t-ratio_1, 27_ =  − 3.07, *P* = 0.03) and marginally greater length gain at multistressor conditions versus ambient conditions (Bonferroni EMMs post-hoc test, t-ratio_1, 27_ =  − 2.37, *P* = 0.07) and at elevated *p*CO_2_ than at elevated temperature (Bonferroni EMMs post-hoc test, t-ratio_1, 27_ =  − 2.19, *P* = 0.07; Fig. 2b). Shell width gain (% change) differed between treatments (ANCOVA, F_1, 27_ = 8.01, *P* = 0.01; Table 2) with significantly greater gains in shell width at elevated *p*CO_2_, both alone and in combination with elevated temperature, than at elevated temperature alone (Bonferroni EMMs post-hoc test, t-ratio_1, 27_ =  − 3.61, *P* = 0.01 and t-ratio_1, 27_ =  − 2.52, *P* = 0.03, respectively) and at elevated *p*CO_2_ than at ambient conditions (Bonferroni EMMs post-hoc test, t-ratio_1, 27_ =  − 2.83, *P* = 0.03; Fig. 2c). Shell height gain (% change) differed between treatments (ANCOVA, F_1, 27_ = 4.85, *P* = 0.04; Table 2), with marginally greater gain in shell height at elevated *p*CO_2_, both alone and in combination with elevated temperature, than at elevated temperature alone (Bonferroni EMMs post-hoc test, t-ratio_1, 27_ =  − 2.75, *P* = 0.06 and t-ratio_1, 27_ =  − 2.30, *P* = 0.07, respectively) and at elevated *p*CO_2_ than at ambient conditions (Bonferroni EMMs post-hoc test, t-ratio_1, 27_ =  − 2.20, *P* = 0.07; Fig. 2d). Ornamentation width gain (% change) differed between treatments (ANCOVA, F_1, 27_ = 5.66, *P* = 0.02; Table 2), with significantly greater ornamentation width gain at elevated *p*CO_2_, both alone and in combination with elevated temperature, than at ambient conditions (Bonferroni EMMs post-hoc test, t-ratio_1, 27_ =  − 2.38, *P* = 0.05 and t-ratio _1, 27_ =  − 2.21, *P* = 0.05, respectively) or at elevated temperature alone (Bonferroni EMMs post-hoc test, t-ratio_1, 27_ =  − 2.49, *P* = 0.05 and t-ratio_1, 27_ =  − 2.31, *P* = 0.05, respectively; Fig. 2e).

### Shell mineralogy

Concentrations of elemental arsenic ([^75^As]) were always below limits of detection and were therefore not included in any further analyses. For the majority of other mineralogical variables, we observed strong (R^2^ ≥ 0.82) and significant pairwise correlations (N = 28 pairwise comparisons; Pearson product-moment correlation, all *P* < 0.001) within *T. squamosa* exoskeleton (i.e., shell and scute together) under ambient seawater conditions (Fig. S1a). However, the number and strength of these correlations decreased under elevated temperature (N = 13 significant correlations; Fig. S1b) and elevated *p*CO_2_ (N = 16 significant correlations; Fig. S1c). Under multistressor conditions, we recovered more significant pairwise correlations than under either stressor individually (N = 25 significant comparisons; Fig. S1d).

**Figure 3:**
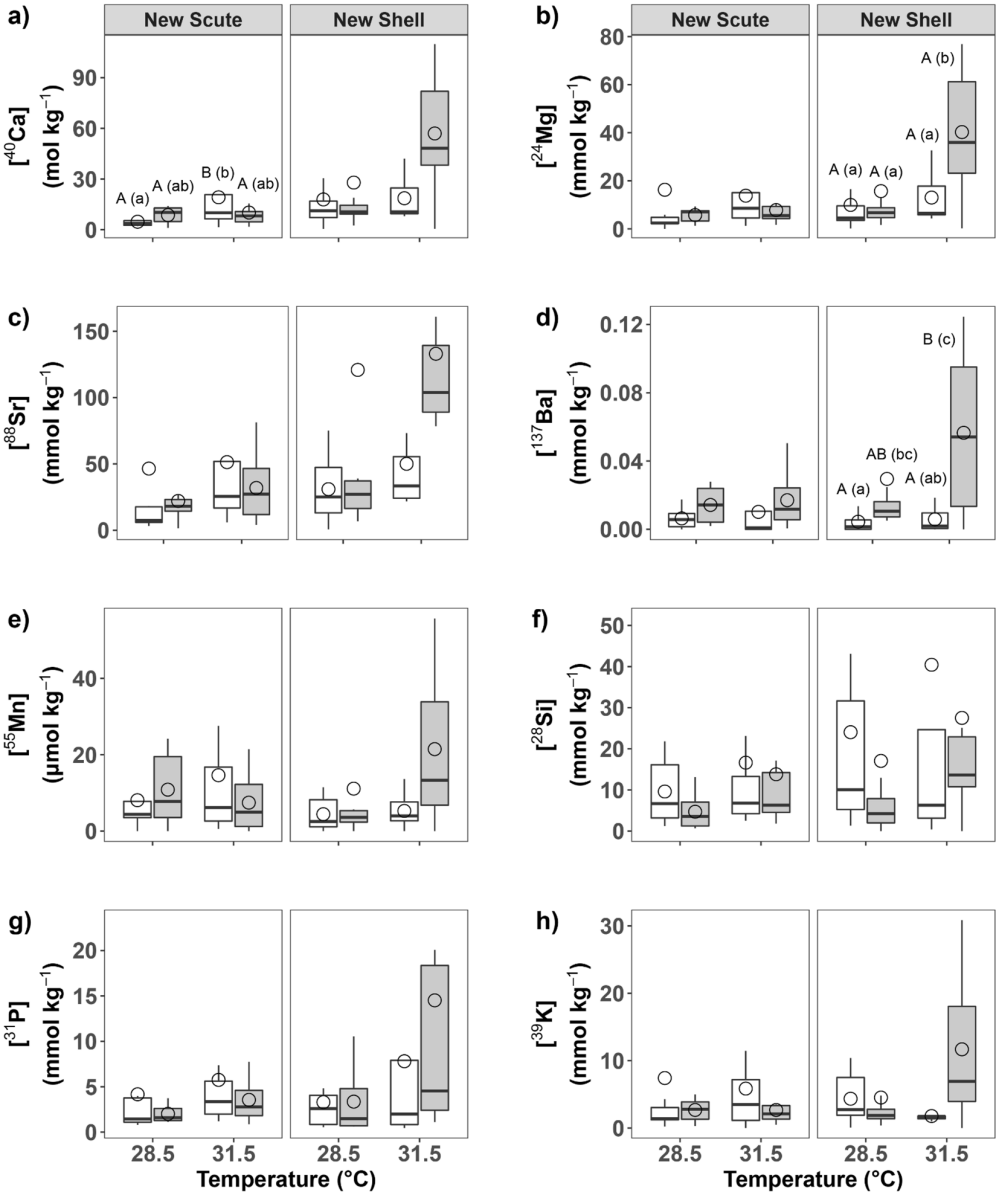
The effects of 60-days exposure to elevated temperature (28.5 and 31.5 ℃) and *p*CO_2_ (450 and 950 µatm) on mineral content (ionic concentrations) of newly-formed shell and scute in *T. squamosa*: (**a**) Calcium, (**b**) Magnesium, (**c**) Strontium, (**d**) Barium, (**e**) Manganese, (**f**) Silicon, (**g**) Phosphorus, and (**h**) Potassium. Boxplots display group means (dots), medians (horizontal dark bar), and interquartile (upper and lower box horizontal lines) and 1.5× interquartile ranges (whiskers). White boxes depict traits measured at 450 µatm *p*CO_2_ and grey at 950 µatm *p*CO_2_. Significantly (P ≤ 0.05) and marginally (0.05 < P ≤ 0.1) different means, according to estimated marginal means (EMMs) tests with FDR-Bonferroni correction, are indicated by different upper- and lowercase letters, respectively.

In newly-formed scute, we observed a significant effect of temperature (ANCOVA, F_1, 25_ = 8.05, *P* = 0.01) on [^40^Ca] with higher [^40^Ca] in scute formed at elevated temperature than at ambient conditions (Bonferroni EMMs post-hoc test, t-ratio_1, 25_ =  − 2.84, *P* = 0.05; Table 3, Fig. 3a). In addition, we observed a marginal interactive effect between temperature and *p*CO_2_ on [^40^Ca] in newly-formed scute (ANCOVA, F_1, 26_ = 3.04, *P* = 0.09) although subsequent post-hoc tests failed to reveal any differences between pairwise comparisons (Bonferroni EMMs post-hoc tests, all *P* > 0.1; Table 3). We observed no other significant or marginal effects of seawater treatments on any other minerals in newly-formed scute (ANCOVAs, all *P* > 0.1; Table S5, Fig. 3b–h).

In newly-formed shell, we observed a significant effect of *p*CO_2_ (ANCOVA, F_1, 26_ = 4.62, *P* = 0.04) on [^137^Ba]. Shells formed at elevated *p*CO_2_, either alone or in combination with elevated temperature, displayed higher [^137^Ba] than shells formed under ambient conditions (Bonferroni EMMs post-hoc tests, t-ratio_1, 26_ =  − 2.15, *P* = 0.08 and t-ratio_1, 26_ =  − 3.47, *P* = 0.01, respectively; Table 3, Fig. 3d). In addition, shells formed under multistressor conditions displayed higher [^137^Ba] than shells formed under elevated temperature alone (Bonferroni EMMs post-hoc test, t-ratio_1, 25_ =  − 3.23, *P* = 0.01; Table 3, Fig. 3d). We also observed a marginal interactive effect between temperature and *p*CO_2_ on [^39^ K] in newly-formed shell (ANCOVA, F_1, 26_ = 3.76, *P* = 0.06) although subsequent post-hoc tests failed to reveal any differences between pairwise comparisons (Bonferroni EMMs post-hoc test, all *P* > 0.1; Table 3). We observed no other significant or marginal effects of seawater treatments on any other minerals in newly-formed shell (ANCOVAs, all *P* > 0.1; Table S6, Fig. 3a–c,e–h).

In older-growth scute, we observed a significant effect of *p*CO_2_ on [^137^Ba] (ANCOVA, F_1, 23_ = 5.00, *P* = 0.03) and a marginal effect of *p*CO_2_ on [^88^Sr] (ANCOVA, F_1, 23_ = 3.65, *P* = 0.07). However, subsequent post-hoc tests failed to reveal any differences between pairwise comparisons for either mineral (Bonferroni EMMs post-hoc test, all *P* > 0.1; Table 4). We observed no other significant or marginal effects of seawater treatments on any other minerals in older-growth scute (ANCOVAs, all *P* > 0.1; Table S7, Fig. S2a–f).

In older-growth shell, we observed a significant effect of temperature (ANCOVA, F_1, 26_ = 6.18, *P* = 0.02) and a marginal effect of the interaction of temperature and *p*CO_2_ (ANCOVA, F_1, 26_ = 5.72, *P* = 0.02) on [^24^Mg] with marginally higher [^24^Mg] under ambient conditions than at elevated temperature (Bonferroni EMMs post-hoc test, t-ratio_1, 26_ = 2.49, *P* = 0.09; Table 4, Fig. S2b). We also observed a significant effect of temperature and of the interaction of *p*CO_2_ and temperature on [^28^Si] (ANCOVAs, F_1, 26_ = 6.23, *P* = 0.02 and F_1, 26_ = 4.81, *P* = 0.04, respectively), [^31^P] (ANCOVAs, F_1, 26_ = 5.40, *P* = 0.03 and F_1, 26_ = 5.10, *P* = 0.03, respectively), and [^39^K] (ANCOVAs, F_1, 26_ = 6.22, *P* = 0.02 and F_1, 26_ = 5.27, *P* = 0.03, respectively). However, subsequent post-hoc tests failed to reveal any differences between pairwise comparisons for these minerals (Bonferroni EMMs post-hoc tests, all *P* > 0.1; Table 4). We observed a marginal effect of temperature (ANCOVA, F_1, 26_ = 3.91, *P* = 0.06) and a significant interactive effect of temperature and *p*CO_2_ (ANCOVA, F_1, 26_ = 4.67, *P* = 0.04) on [^40^Ca], but subsequent post-hoc tests failed to reveal any differences between pairwise comparisons for either effect (Bonferroni EMMs post-hoc tests, all *P* > 0.1; Table 4). There was a marginal effect of the interaction of temperature and *p*CO_2_ (ANCOVA, F_1, 26_ = 3.70, *P* = 0.07) on [^137^Ba] although subsequent post-hoc tests failed to reveal any differences between pairwise comparisons (Bonferroni EMMs post-hoc test, all *P* > 0.1; Table 4). Finally, we also observed significant effects of temperature (ANCOVA, F_1, 26_ = 7.75, *P* = 0.01), *p*CO_2_ (ANCOVA, F_1, 26_ = 4.61, *P* = 0.04), and the interaction of temperature and *p*CO_2_ (ANCOVA, F_1, 26_ = 6.98, *P* = 0.01) on [^55^Mn]. However, subsequent post-hoc tests revealed significantly higher [^55^Mn] only at elevated temperature as compared to under ambient conditions (Bonferroni EMMs post-hoc test, t-ratio_1, 26_ = 2.78, *P* = 0.06; Table 4, Fig. S2e). We observed no other significant or marginal effects of seawater treatments on any other minerals in older-growth shell (ANCOVAs, all *P* > 0.1; Table S8, Fig. S2a–f).

**Figure 4:**
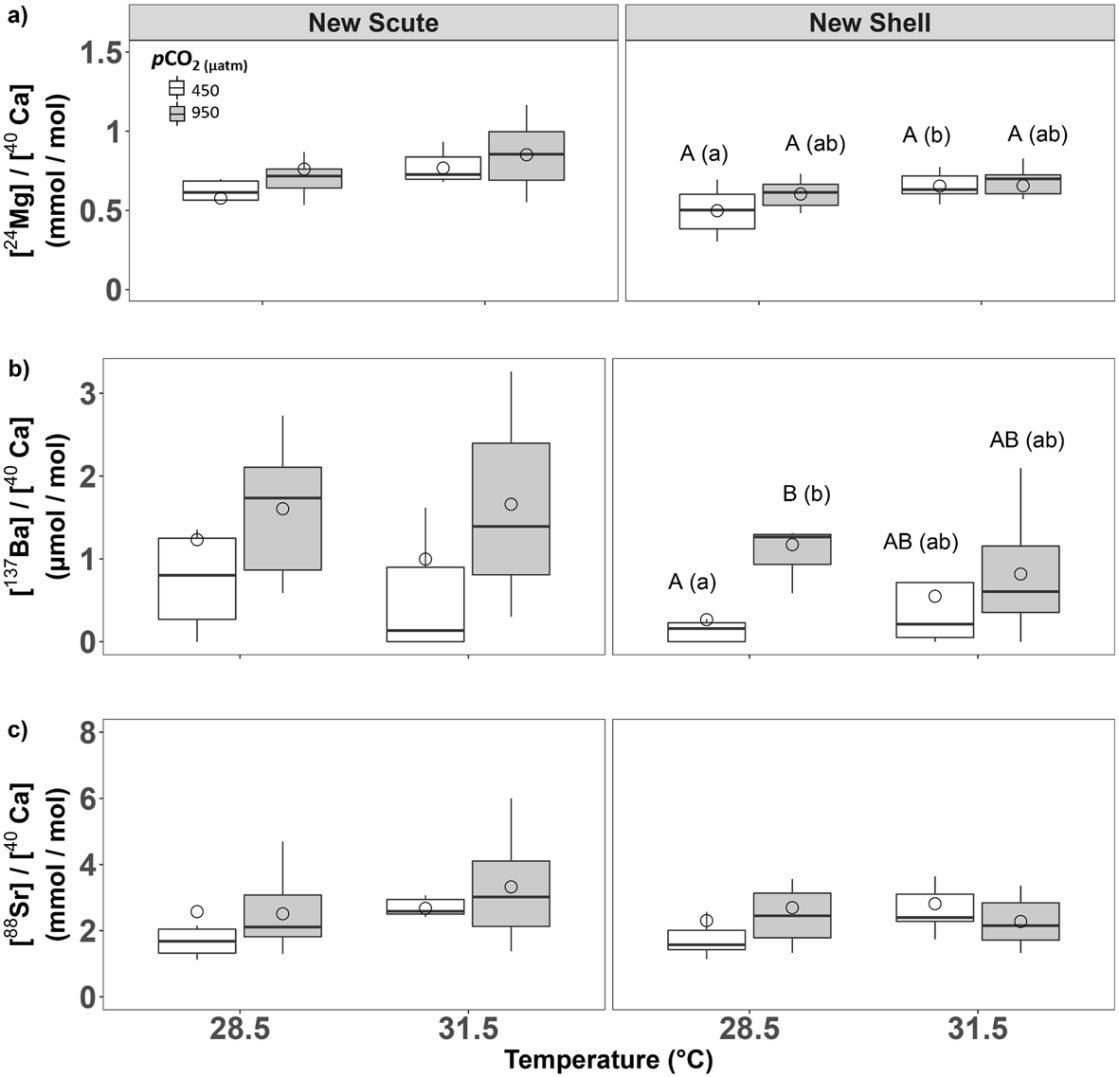
The effects of 60-days exposure to elevated temperature (28.5 and 31.5 ℃) and *p*CO_2_ (450 and 950 µatm) on select element/calcium ratios in *T. squamosa* newly-formed shell and scute: (**a**) Mg/Ca ratio, (**b**) Ba/Ca ratio, and (**c**) Sr/Ca ratio. Boxplots display group means (dots), medians (horizontal dark bar), and interquartile (upper and lower box horizontal lines) and 1.5× interquartile ranges (whiskers). White boxes depict traits measured at 450 µatm *p*CO_2_ and grey at 950 µatm *p*CO_2_. Significantly (*P* ≤ 0.05) and marginally (0.05 < *P* ≤ 0.1) different means, according to estimated marginal means (EMMs) tests with FDR-Bonferroni correction, are indicated by different upper- and lowercase letters, respectively.

Select Group II elements (i.e., ^24^Mg, ^88^Sr, and ^137^Ba) were also examined as ratios with [^40^Ca] as the denominator. Because of their similarly-sized ionic radii and electrochemical properties, these cations are known to substitute for ^40^Ca within the calcium carbonate matrix of bivalve shells^11,41^ with ^24^Mg replacing ^40^Ca in calcite^42^ and ^88^Sr and ^137^Ba replacing ^40^Ca in aragonite^43^. In newly-formed scute, we observed no effect of seawater treatments on any element/Ca ratios (ANCOVAs, all *P* > 0.1; Table S5). However, in newly-formed shell, we observed a significant effect of elevated temperature (ANCOVA, F_1, 26_ = 7.38, *P* = 0.01) on [^24^Mg]/[^40^Ca] with marginally higher [^24^Mg]/[^40^Ca] at elevated temperature than under ambient conditions (Bonferroni EMMs post-hoc test, t-ratio_1, 26_ =  − 2.72, *P* = 0.07; Table 3, Fig. 4a). We also observed a significant effect of elevated *p*CO_2_ (ANCOVA, F_1, 26_ = 8.98, *P* < 0.01) on [^137^Ba]/[^40^Ca] with higher [^137^Ba]/[^40^Ca] at elevated *p*CO_2_ than under ambient conditions (Bonferroni EMMs post-hoc test, t-ratio_1, 26_ =  − 3.00, *P* = 0.04; Table 3, Fig. 4b). We observed no effects of seawater treatments on [^88^Sr]/[^40^Ca] in newly-formed shells (ANCOVAs, all *P* > 0.1; Table S6, Fig. 4c). In older-growth scute, we observed no effect of seawater treatments on any element/Ca ratios (ANCOVAs, all *P* > 0.1; Table S7, Fig. S3a–c). However, in older-growth shell, we observed a significant effect of elevated temperature (ANCOVA, F_1, 26_ = 5.16, *P* = 0.03) on [^24^Mg]/[^40^Ca] with lower [^24^Mg]/[^40^Ca] under multistressor conditions than under ambient conditions (Bonferroni EMMs post-hoc test, t-ratio_1, 26_ = 2.97, *P* = 0.03; Table 4, Fig. S3a). We observed no effects of seawater treatments on any other element/Ca ratios in older-growth shells (ANCOVAs, all *P* > 0.1; Table S8, Fig. S3b,c).

### Shell organic content

We observed a marginal effect of elevated temperature (ANCOVA, F_1, 20_ = 3.10, *P* = 0.09) on N-content (weight%) in newly-formed shell. However, subsequent post-hoc testing failed to reveal any differences between pairwise comparisons (Bonferroni EMMs post-hoc test, all *P* > 0.1; Table 2). We observed no effects of seawater treatments on any other organic content variable in newly-formed shells (ANCOVAs, all *P* > 0.1; Table S4).

## Discussion

Increasing atmospheric *p*CO_2_ is driving reduced carbonate bioavailability and elevated sea surface temperatures (SSTs) throughout the World’s oceans. These drivers have the potential to impact shell formation in ecologically and economically important calcifying species such as giant clams. Our results help elucidate the extent to which ocean warming and acidification, alone or in concert, influence shell growth rates and shell mineral and organic content in juvenile *Tridacna squamosa* giant clams. Overall, we show that alterations in seawater *p*CO_2_, similar to those projected to occur with the progression of ocean acidification this century, have a stronger influence on shell mineralogy in juvenile *T. squamosa* than shifts in temperature, projected to occur with ongoing ocean warming, and highlight the importance of seawater *p*CO_2_ (and thus pH) as a driver of not only calcification rates but also of mineral and organic content in giant clam shells.

### Shell morphometry and growth

Contrary to our initial hypotheses, we observed no impact of elevated temperature and a positive impact of elevated *p*CO_2_ on shell growth in juvenile *T. squamosa*. Clams in our study displayed, on average, 2.5x and 1.7x greater gains in shell length and width, respectively, under elevated *p*CO_2_ than under ambient conditions. These results were surprising in light of the majority of previous studies in giant clams that have indicated that elevated SSTs and *p*CO_2_ have positive effects on growth^44^ and inhibitory effects on calcification^22,45,46^, respectively.

For example, thermally-enhanced growth has been observed in many tridacnid species: including in adult *T. squamosa* from the Red Sea^44^, as well as in *Tridacna squamosina*^44^, *Tridacna crocea*^11,47^, *Tridacna derasa*^47^, *Tridacna maxima*^44,47,48^, *Tridacna gigas*^49^, and *Hippopus hippopus*^50^. However, detrimental effects have also been observed at extremely elevated SSTs in some instances. In these cases, it is likely that SSTs approached or surpassed ecologically-relevant thermal thresholds resulting in deleterious impacts on growth. For example, in *H. hippopus* clams, extended exposure to SSTs > 27 °C resulted in a period of erratic growth (i.e., high intra-individual variation) followed by decreased calcification rates^50^. Similarly, a study of gene expression profiles in juvenile *T. maxima* exposed to SSTs of 32 °C for approx. one week showed that clams upregulated genes involved in the scavenging of reactive oxygen species and in fatty acid rearrangement likely in response to heat stress^18^. Finally, in juvenile *T. squamosa*, exposure to SSTs > 30 °C for over 40 days led to significantly increased mortality—although low irradiance may also have contributed to this effect^21^. In this study, we did not observe increased mortality under elevated temperature. However juveniles were exposed to relatively severe SST conditions (+ 3 °C over summer maximum temperatures) which may have been at or near their upper thermal limits for growth. This could explain why we observed no thermal-enhancement of shell growth rates.

In contrast to the effects of elevated temperature, previous investigations of the effects of elevated *p*CO_2_ on calcification in giant clams have largely reported decreased shell extension rates under ocean acidification conditions^13,22,45,46,51^. Reduced shell growth rates have been reported previously in juvenile *T. squamosa* clams (+ 350/1000 µatm for one year^46^ and + 585/885 µatm for 10 weeks^51^) as well as in juveniles of the closely related species *T. maxima* under elevated *p*CO_2_ (+ 800 μatm for 9 weeks^13^). However, conflicting evidence exists for *T. crocea* in which a significant reduction in shell height gain was observed under elevated *p*CO_2_ (+ 600/+ 1600 μatm for 4 weeks), but shell length gain remained unimpacted in the same individuals^23^. Similarly, no effect of *p*CO_2_ on shell growth rate was observed in either *T. crocea* or *T. squamosa* clams of the same life stage under similar conditions (+ 350/+ 1000 μatm for one year^46^ and + 788 µatm for 6 weeks^52^, respectively). Finally, shell extension rates of juvenile *T. derasa* clams increased in exposure to high-nutrient, high-*p*CO_*2*_ conditions^46^. Thus, although the majority of studies report negative effects of elevated *p*CO_2_ on giant clam calcification rates, these effects appear to have been offset in some species under some conditions. In this study, we observed enhanced shell growth rates in juvenile *T. squamosa* under elevated *p*CO_2_.

At the molecular level, prolonged exposure of giant clams to elevated *p*CO_2_ reduces net calcification rate presumably as a result of decreased carbonic anhydrase activity^52^. However, elevated *p*CO_2_ can increase calmodulin activity in giant clams^52^, and this calcium-binding protein is increasingly recognized as an important contributor to calcium precipitation in bivalves^52–54^. Thus, one possible explanation for the positive effects of ocean acidification on shell growth rates we observed in *T. squamosa* in this study may be an increase in calmodulin-driven calcium-binding/precipitation under elevated *p*CO_2_ conditions sufficient enough to promote shell growth as has recently been reported in the Pacific oyster *Crassostrea gigas*^54^.

Another possible explanation for the positive effects of ocean acidification on growth rates in *T. squamosa* may be a result of indirect fertilization through increased supply of inorganic carbon for symbiont photosynthesis. Peak photosynthetic rates in isolated zooxanthellae maintained under conditions similar to those within the giant clam hemolymph were found to be significantly less than the theoretical maximum, and symbionts, *in hospite,* are capable of completely depleting inorganic carbon from the host hemolymph^16,55^. This indicates that zooxanthellae are partially carbon limited within their host^56^. In symbiotically-intact giant clams, supply of inorganic carbon to the zooxanthellae is enhanced by the host through an efficient carbon-concentration mechanism^57,58^. Exposure to elevated *p*CO_2_ may therefore increase the rate of carbon supply to the zooxanthellae by elevating levels of inorganic carbon in host hemolymph (i.e., tissue acidosis) and increasing export of photosynthetic products to the clam thus fueling host growth. However, this carbon enrichment hypothesis remains equivocal (discussed further in the shell organic content section, below).

In addition to the potential for elevated *p*CO_2_ to increase host growth rates, giant clams are also well known to exhibit light-enhanced calcification indicating that increased symbiont photosynthesis also drives increased host biomineralization^11,49,59–62^. In this way, giant clams may be able to leverage increased environmental inorganic carbon to promote symbiont photosynthesis, benefiting from increased availability of O_2_ and carbohydrates, as well as increased rates of carbonate precipitation. Under this proposed mechanism, exposure to moderately elevated seawater *p*CO_2_ could actually be beneficial for tridacnid clams and could explain the positive effects on growth we report in juvenile *T. squamosa* clams. However, recent studies in juvenile *T. maxima* and *T. squamosa* indicated that exposure to elevated *p*CO_2_ (ca. + 800 µatm) resulted in significant declines in symbiont photosynthetic yield accompanied by reduced zooxanthellae density^13,52^ whereas, in juvenile *T. crocea*, zooxanthellae density increased under elevated *p*CO_2_ but photosynthetic productivity remained constant thus suggesting reduced output per algal cell^23^. Thus, this “fertilization effect” may not be universal across populations, species, and/or environments. For example, the effectiveness of this putative “carbon pump” likely also depends on ambient irradiance levels^22^, as well as on a concomitant increase in the availability of N to the *zooxanthellae* (see our discussion of shell organic content, below). Differences in rearing conditions, particularly those contributing to photosynthetic productivity, may therefore partially explain the discrepancies in shell growth responses to elevated *p*CO_2_ in giant clams as reported above.

### Shell mineralogy

#### Elemental concentrations

With the exception of [^137^Ba] and [^24^Mg] in newly-formed shell, discussed in the element/Ca section below, and [^40^Ca] in newly-formed scute, which increased at elevated temperature, mean concentrations of trace elements within *T. squamosa* scutes or shell were not impacted by exposure to either elevated temperature or *p*CO_2_ (Fig. 3). However, under multistressor conditions, inter-individual variation in trace mineral concentrations within newly-formed shell increased. This increased variability in trace mineral incorporation mirrors the increased intra-individual variation in calcification rates reported in *H. hippopus* under elevated temperature, which was interpreted as a signal of the onset of thermal stress^50^. Similarly, we hypothesize that the increased variation in trace mineral content we observed in *T. squamosa* shells formed under multistressor conditions may also indicate the existence of a physiological tolerance threshold after which clams exhibit increased idiosyncrasy in response deriving from different sensitivities between individuals (see our discussion of organismal stress below). This implies that reported variations in mineral and isotopic content of giant clam shells between individuals at the same site^5^ may be driven, at least in part, by differential physiological susceptibility to different and/or different combinations of environmental drivers. This observation may have important implications for the use of giant clam carbonates as paleoclimate proxies and for paleoclimate reconstructions, especially when those reconstructions are predicated from one, or a few, individual(s).

#### Element/Ca ratios

With the exception of [^75^As], all mineralogical response variables positively covaried under ambient conditions (Fig. S1a). However, the number and strength of these correlations decreased under elevated temperature (Fig. S1b) and elevated *p*CO_2_ (Fig. S1c) in isolation, but not in combination (Fig. S1d). These results suggest a potential antagonistic effect of simultaneous exposure to elevated temperature and *p*CO_2_ on shell trace mineral incorporation, and thus a potential buffering of shell mineral structure under multistressor conditions.

Significant attention has been devoted to use of specific element/Ca ratios as paleoclimate-indices—in particular [^88^Sr]/[^40^Ca], [^24^Mg]/[^40^Ca], and [^137^Ba]/[^40^Ca]. However, the exact relationship between SST and these elemental ratios in giant clams’ shells remains unresolved. In the case of shell [^88^Sr]/[^40^Ca], both positive- and non-correlations with temperature have been reported across a variety of timescales (Table S1). Similarly, positive relationships between shell [^24^Mg]/[^40^Ca] and SST have been reported in several tridacnid species, though counterexamples exist as well (Table S1). Our results from modern *T. squamosa* juveniles are also somewhat equivocal. Although we observed no effect of elevated temperature on shell [^88^Sr]/[^40^Ca] or [^137^Ba]/[^40^Ca] we did observe a strong positive relationship between temperature and [^24^Mg]/[^40^Ca] in newly-formed scute and shell (Fig. 4a). In addition, we observed a significant increase in [^137^Ba]/[^40^Ca] ratios in newly-formed *T. squamosa* shell under elevated *p*CO_2_ (Fig. 4b) suggesting an increased substitution of ^137^Ba in place of ^40^Ca in the CaCO_3_ lattice of the shell. To our knowledge, this is the first report of a significant effect of seawater *p*CO_2_ on any element/Ca ratio in giant clams. Because replacement of ^40^Ca by other Group II elements has effects on the crystalline ultrastructure of shells^63^, it has the potential to alter their mechanical properties as well. For example, *T. squamosa* shells with lower CaCO_3_ content as a proportion of their weight have been demonstrated to suffer from decreased resistance to crushing forces (i.e., reduced compressive strength)^51^. Thus, the increasing prevalence of ^137^Ba we observed in shells at elevated *p*CO_2_ may provide another mechanism for weakening bivalve skeletal structures beyond the effects of reduced calcification and/or shell dissolution generally associated with ocean acidification.

Previous studies have also suggested that increased [^137^Ba]/[^40^Ca] in bivalve shells can result from increased dietary input of ^137^Ba, for example from increased ingestion of phytoplankton containing ^137^Ba^41^. Although we did not measure filtration/clearance rates in this study, experimental aquaria were maintained with pre-filtered seawater and clams were not given any supplemental feedings of plankton. Thus, whereas a shift in the relative balance between photo- and heterotrophy in this species could potentially have contributed to the elevated [^137^Ba]/[^40^Ca] we observed in shells formed under multistressor conditions, it was likely not the primary driver. In contrast, previous work in foraminiferans and corals has identified a strong relationship between carbonate [^137^Ba]/[^40^Ca] and increased freshwater input^64–68^ and/or upwelling of deep, nutrient-rich water^40,69–74^. Interestingly, both processes are likely to temporarily alter local or micro-habitat pH and carbonate saturation levels. In combination with our findings, these data suggest that bivalve carbonate [^137^Ba]/[^40^Ca] might be responsive to, and therefore a useful proxy for, environmental *p*CO_2_. This also has important implications for using giant clams as paleoclimate indices as increases in seawater *p*CO_2_ (including past events) could potentially result in the same [^137^Ba]/[^40^Ca]/Ca signals as those caused by decreases in SST (see the relationship summaries in Table S1).

### Shell organic content

**Figure 5:**
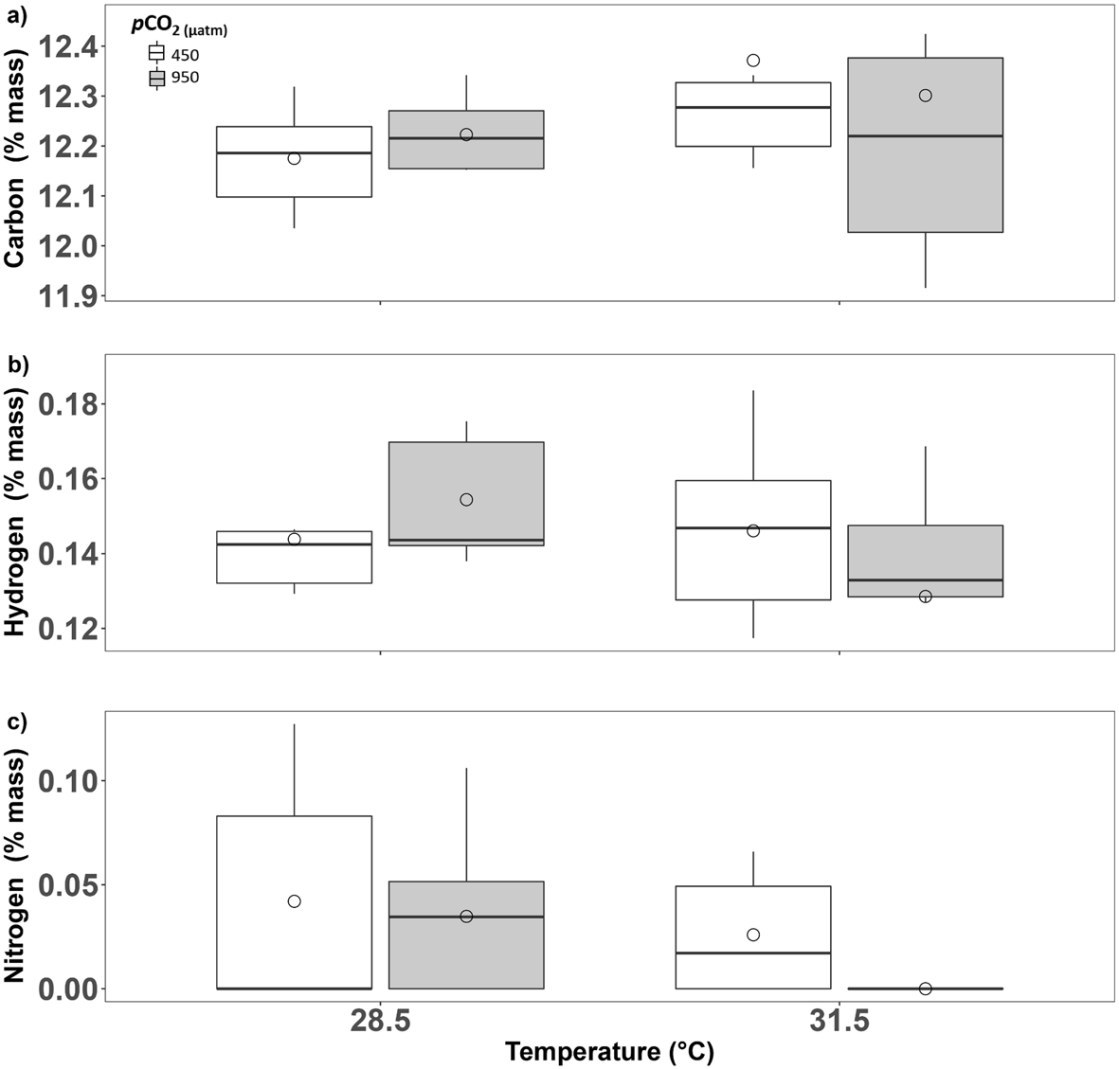
The effects of 60-days exposure to elevated temperature (28.5 and 31.5 ℃) and *p*CO_2_ (450 and 950 µatm) on organic content (weight %) of *T. squamosa* shell: (**a**) % carbon, (**b**) % hydrogen, and (**c**) % nitrogen. Individual data (circles) are presented alongside group means (diamonds). Boxplots display group means (dots), medians (horizontal dark bar), and interquartile (upper and lower box horizontal lines) and 1.5× interquartile ranges (whiskers). White boxes depict traits measured at 450 µatm *p*CO_2_ and grey at 950 µatm *p*CO_2_.

Although it represents only a small fraction of the molluscan shell in terms of weight (typically < 5% in bivalves, ca. 0.9% in *Tridacna derasa*^75^), the shell organic matrix plays a significant role in determining shell crystalline microstructure^76^. We observed no effect of exposure to elevated temperature and *p*CO_2_ on C- (total organic and inorganic) or H-content of *T. squamosa* shells (Fig. 5a,b). However, under multidriver conditions, only a single clam displayed measurable shell organic N after 60 days of exposure (Fig. 5c) and this individual was identified as a data outlier. Analysis of the N-content data without this individual revealed an emerging pattern of decreasing shell organic nitrogen content under elevated temperature conditions in juvenile *T. squamosa* clams with a potentially synergistic, negative, impact of elevated SST and *p*CO_2_ (Fig. 5c). However, to further test the significance of this trend, a larger number of observations may be needed.

As the organic matrix of tridacnine shell is primarily composed of polysaccharides and glycosylated and un-glycosylated proteins and lipids^75^, we postulate that the relative decrease in nitrogen content of shells formed under multistressor conditions may reflect increasing competition for nitrogen between the algal symbiont (as a result of increased primary production from increased inorganic carbon supply) and the host (lipoprotein biosynthesis). There is substantial evidence that symbiotic zooxanthellae are nitrogen-limited *in hospite* in giant clams and that nitrogen-enrichment and eutrophication can enhance host growth^44,77–82^. Additionally, exposure to elevated seawater *p*CO_2_ (ca. 30 days) was shown to enhance O_2_ production in clam-hosted zooxanthellae, and zooxanthellae photosynthetic yield was higher under multistressor conditions (after 30-days exposure) than under ambient conditions thus implying *p*CO_2_-driven fertilization of giant clam growth^13^. However, conflicting evidence also exists showing reduced photosynthetic yield of clam-hosted zooxanthellae^23^ or, alternatively, increased photosynthetic rates *per* individual zooxanthellae, but overall decreased algal density and net photosynthetic productivity at elevated *p*CO_2_^52^. We postulate that some of this discrepancy regarding the fertilizing effect of elevated *p*CO_2_ on giant clam growth may stem from the interplay of several factors contributing to zooxanthellae photosynthesis *in hospite*, including irradiance and nutrient supply. For example, if elevated seawater *p*CO_2_ serves as a potential enhanced inorganic carbon source for the algal symbionts in giant clams, as discussed previously, then this would necessitate a concomitant increase in supply of inorganic nitrogen. Given that we see a trend towards nitrogen-depletion in giant clam shells under elevated *p*CO_2_, especially in combination with elevated temperature, our data may provide additional indirect evidence for increased symbiont productivity under elevated *p*CO_2_. This in turn ultimately reduces the amount of nitrogen that is available for incorporation into the host shell potentially altering mineral lattice formation. Our data indicate that this putative nitrogen drawdown is more pronounced under multistressor conditions than under elevated *p*CO_2_ alone.

### Organismal stress

Increased shell element/Ca ratios, especially [^24^Mg]/[^40^Ca], are hypothesized to function as a biomarker of organismal stress in giant clams, in addition to a paleo-temperature index^11^. Our data partially support this hypothesis as we observed elevated shell [^24^Mg]/[^40^Ca] under both elevated temperature and multistressor conditions (Fig. 4a). Additionally, we observed increased inter-individual variation in nearly all trace minerals in shells formed under multistressor conditions. Both inter- and intra-individual variation are increasingly recognized as important biological traits underpinning organismal responses to environmental change^83–85^ and increased variance in elemental composition of giant clam shells has also been proposed as a signature of the onset of thermal stress^50^. However, despite these findings, we saw no evidence for negative impacts of exposure to elevated temperature or *p*CO_2_ on shell formation or animal growth (i.e., mass gain) in juvenile *T. squamosa.* To the contrary, we observed positive effects of these drivers on shell growth and mass gain in this species. Thus, our data suggest that elevated [^24^Mg]/[^40^Ca] in giant clam shells is perhaps best interpreted as the result of a physiological phase shift, potentially signaling the mobilization of compensatory mechanisms (i.e., beneficial plasticity as has been reported in *Mytilus* mussels^86^), which may be different or differently modulated in different individuals, rather than the onset of organismal stress sensu stricto.

Interpretation of these data is complex however, especially in light of the fact that element/Ca ratios in giant clam shells can vary significantly between individuals at the same collection site (i.e., high inter-individual variation as described above)^87^ and between different regions of the shell either as a result of variation in deposition across life history stages^4,88^ or as a result of tissue-specific deposition effects^89^. For example, we observed consistently lower variation in the trace mineral content of scute across treatments suggesting that this region of the exoskeleton is likely formed under different constraints from the shell proper. This dampened response in scutes could reflect selective pressures for maintaining the defensive capabilities in these structures and may reduce their utility as a paleoclimate indices.

## Conclusion

We demonstrate that exposure to elevated seawater temperature and *p*CO_2_ altered shell growth rates and composition in juveniles of the fluted giant clam *T. squamosa*. Under elevated seawater temperature, shell [^24^Mg]/[^40^Ca] ratio increased and exposure to elevated *p*CO_2_, alone or in combination with elevated temperature, strongly impacted shell formation, increasing shell growth rate, mass gain, and [^24^ Mg]/[^40^Ca] and [^137^Ba]/[^40^Ca] ratios. Simultaneous exposure to both drivers resulted in increased inter-individual variation in shell mineral composition and reduced organic N-content, which we hypothesize may signal the onset of physiological stress and/or increased competition for N between the clam host and its algal symbionts. As a consequence, it is increasingly clear that an improved understanding of the physiological mechanisms underpinning shell-formation in these unique, photosymbiotic bivalves is necessary. It remains to be determined, however, whether these effects directly or indirectly impact giant clam survival in the long-term or whether these alterations represent compensatory plasticity meant to maintain overall performance, including potential enhancement of photosymbiont productivity, in the face of multiple stressors. For example, a recent review of the effects of elevated *p*CO_2_ on bivalve shell mineralogy suggests a large capacity for beneficial transgenerational plasticity^90^. Our results suggest the onset of altered physiological states (e.g., shifted heterotrophic-autotrophic balance) in giant clams under projected ocean warming and acidification which may ultimately reduce the protective capacities of their shells and alter exoskeleton element/Ca ratios. This latter finding may have important implications for the use of giant clam carbonates as paleoclimate proxies as we find that changes in seawater temperature and seawater *p*CO_2_ (and/or pH) are capable of driving similar shifts in carbonate element/Ca ratios.

## Supplementary Information

Supplementary Information.

## Data Availability

The datasets generated and analyzed in this study are available in the Zenodo repository (10.5281/zenodo.6401552).
