# Supplementary material for: Elevated temperature and carbon dioxide levels alter growth rates and shell composition in the fluted giant clam, Tridacna squamosa
Source: Sci Rep. 2022 Jun 30;12:11034. doi: 10.1038/s41598-022-14503-4 (PMC9247080; doi:10.1038/s41598-022-14503-4)
Supplement: Supplementary file 1 — Supplementary Information. [file 41598_2022_14503_MOESM1_ESM.docx]

# **SUPPLEMENTAL MATERIALS**


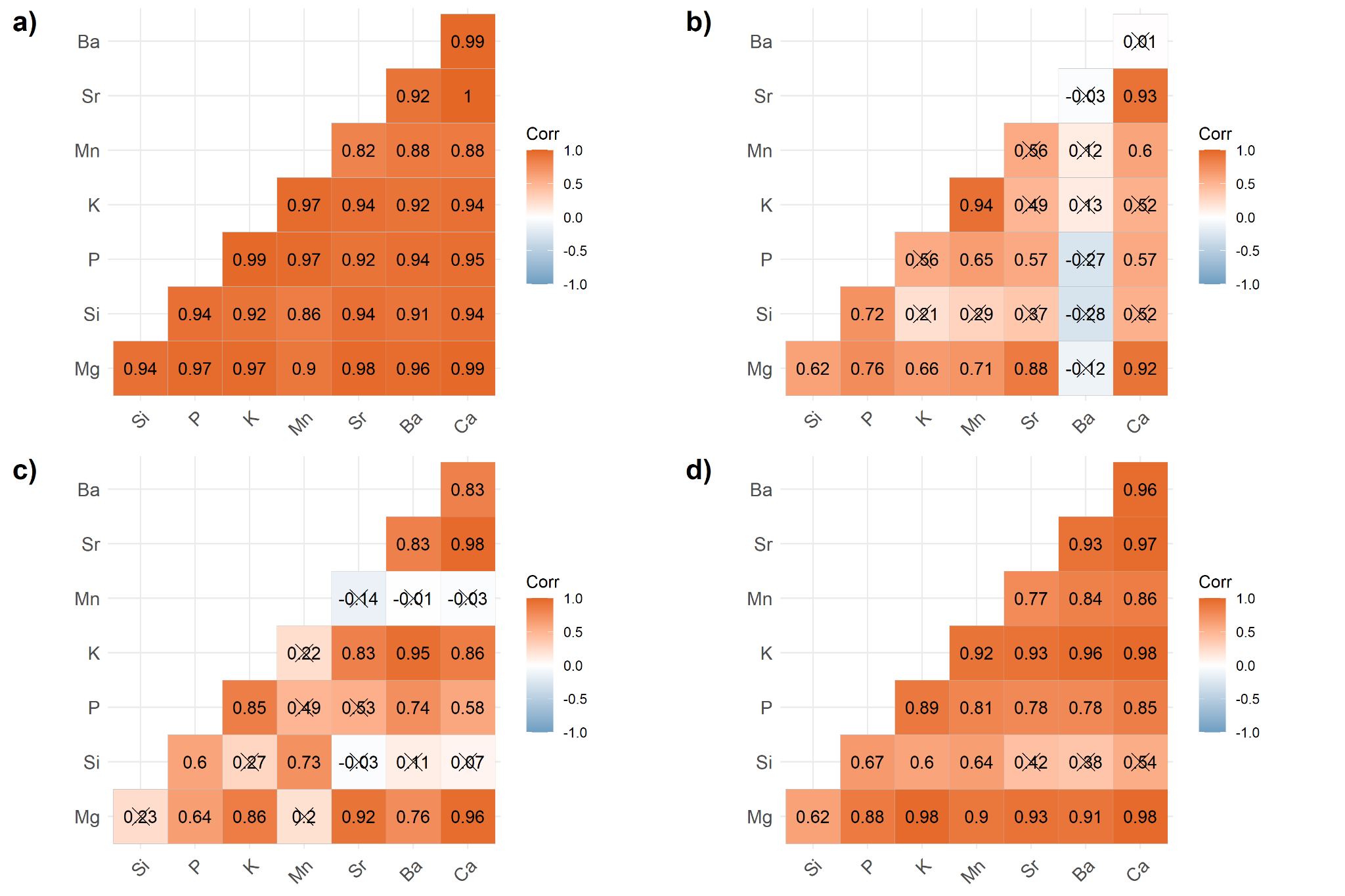


## Supplemental Figure 1.

Correlation coefficients (R^2^-values) of linear regressions between ionic element species in Tridacna squamosa skeleton (both shell and scute) formed after 60 d of exposure to a) ambient conditions, b) elevated temperature, c) elevated pCO_2_, and d) multistressor conditions. Darker colors indicate stronger positive (red) or negative (blue) correlations (e.g. P < 0.001) whereas insignificant correlations are crossed-out.


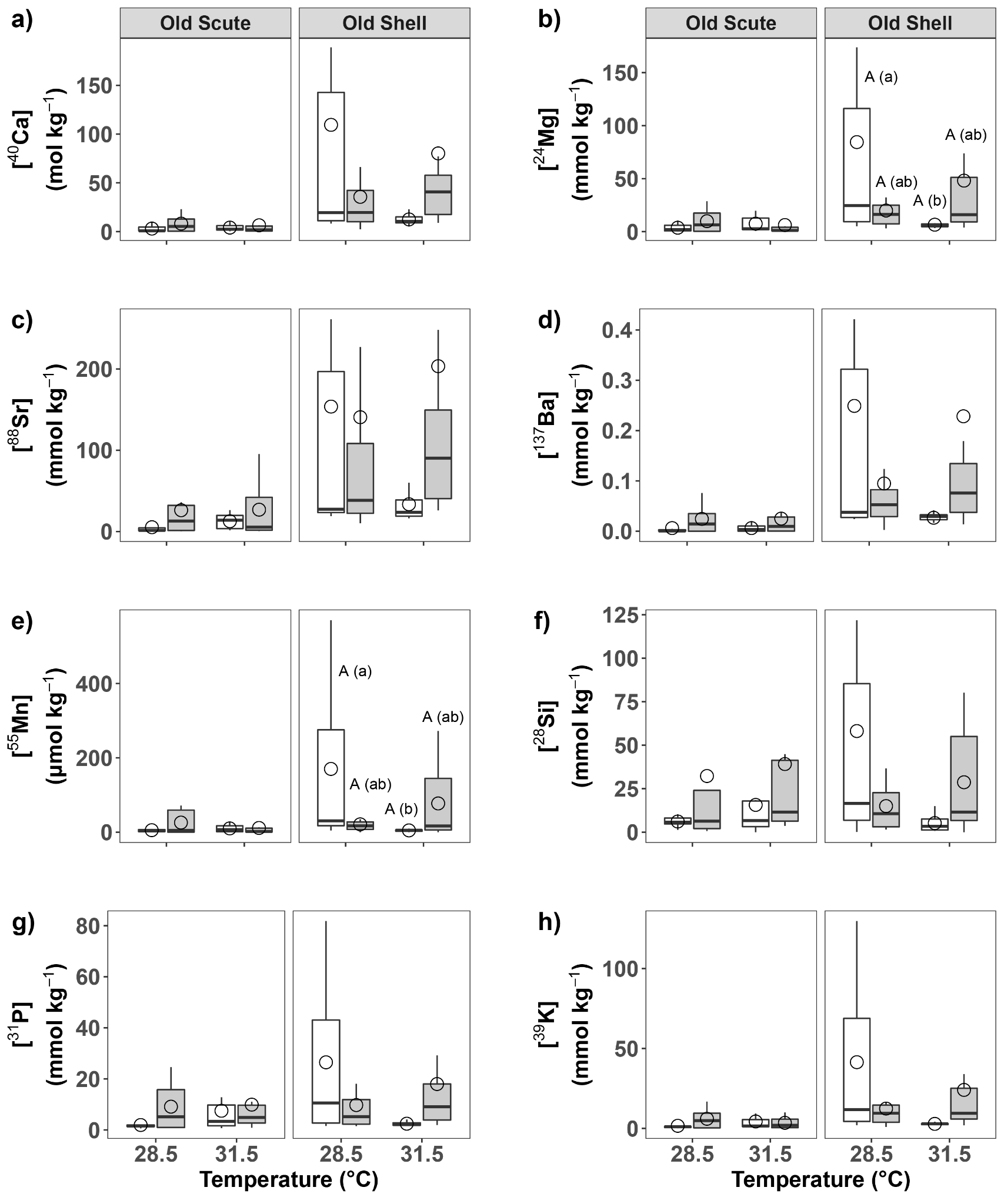


## Supplemental Figure 2.

The effects of 60-d exposure to elevated temperature (28.5 and 31.5 ℃) and *p*CO_2_ (450 and 950 µatm) on mineral content (ionic concentrations) of older-growth shell and scute in *T. squamosa*: (a) Calcium, (b) Magnesium, (c) Strontium, (d) Barium, (e) Manganese, (f) Silicon, (g) Phosphorus, and (h) Potassium. Boxplots display group means (dots), medians (horizontal dark bar), and interquartile (upper and lower box horizontal lines) and 1.5x interquartile ranges (whiskers). White boxes depict traits measured at 450 µatm *p*CO_2_ and grey at 950 µatm *p*CO_2_. Significantly (*P* ≤ 0.05) and marginally (0.05 < *P* ≤ 0.1) different means, according to estimated marginal means (EMMs) tests with FDR-Bonferroni correction, are indicated by different upper- and lowercase letters, respectively.


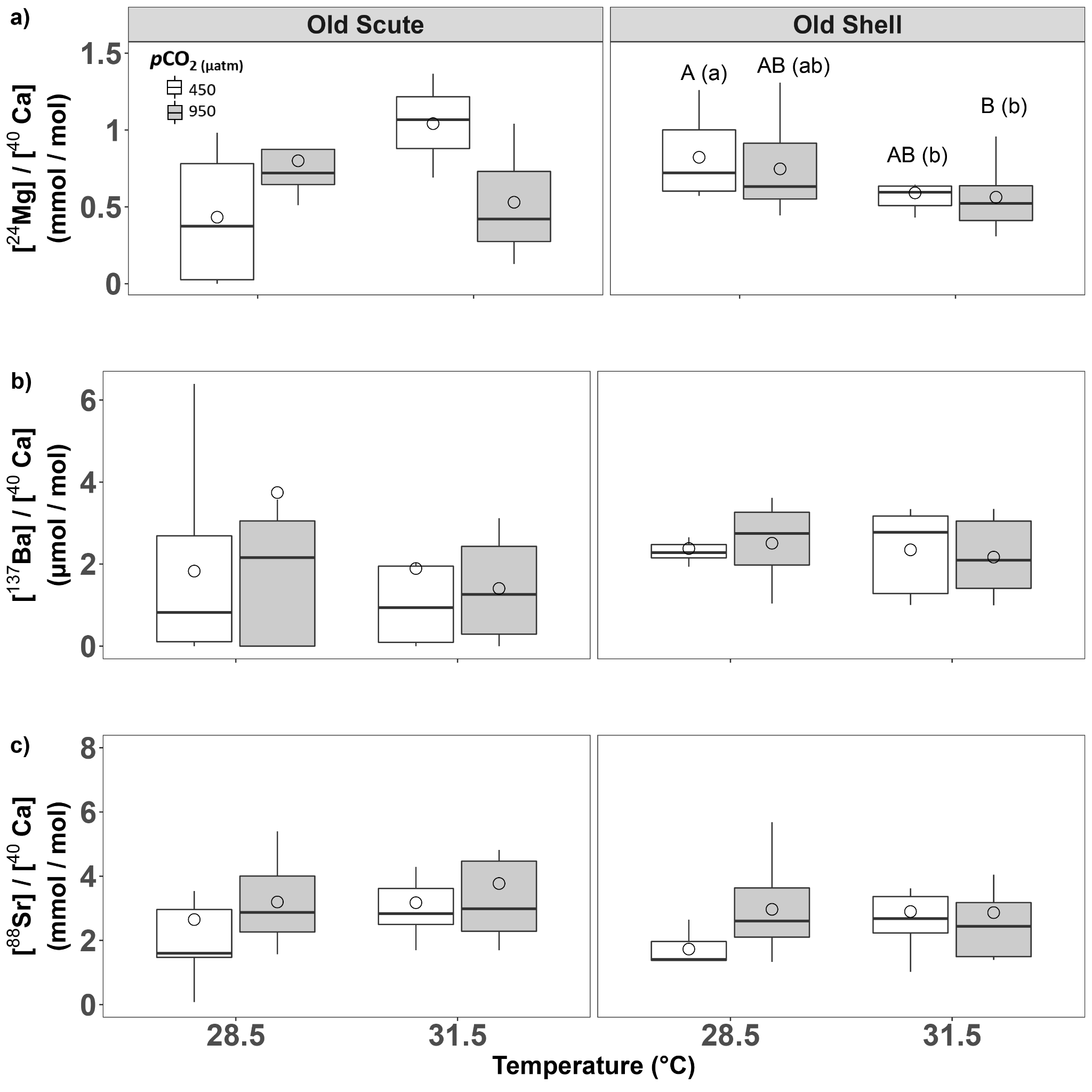


## Supplemental Figure 3.

The effects of 60-d exposure to elevated temperature (28.5 and 31.5 ℃) and *p*CO_2_ (450 and 950 µatm) on select element/calcium ratios in *T. squamosa* older-growth shell and scute: (a) [^24^Mg]/[^40^Ca] ratio, (b) [^137^Ba]/[^40^Ca] ratio, and (c) [^88^Sr]/[^40^Ca] ratio. Boxplots display group means (dots), medians (horizontal dark bar), and interquartile (upper and lower box horizontal lines) and 1.5x interquartile ranges (whiskers). White boxes depict traits measured at 450 µatm *p*CO_2_ and grey at 950 µatm *p*CO_2_. Significantly (*P* ≤ 0.05) and marginally (0.05 < *P* ≤ 0.1) different means, according to estimated marginal means (EMMs) tests with FDR-Bonferroni correction, are indicated by different upper- and lowercase letters, respectively.

##

## Supplemental Table 1.

Literature summary of environmental effects on shell element/Ca ratios in *Tridacna* species. Non-significant relationships denoted by NS.

| **Environmental Driver** | **Mineral** | **Time Scale** | **Relationship** | **Species** |
| --- | --- | --- | --- | --- |
| Temperature | [^88^Sr]/[^40^Ca]  [^24^Mg]/[^40^Ca]  [^137^Ba]/[^40^Ca] | Weekly  Seasonal  Seasonal  Seasonal  Seasonal  Seasonal  Weekly  Seasonal  Seasonal  Monthly  Weekly  Seasonal  Seasonal  Seasonal  Weekly  Seasonal  Seasonal  Monthly  Weekly  Seasonal  Weekly  Seasonal  Seasonal  Seasonal  Monthly | Anticorrelated  NS  Anticorrelated  Anticorrelated  NS  NS  NS  Equivocal*  Weakly Correlated  NS  Anticorrelated  Equivocal*  Correlated  Correlated  NS  Correlated  Correlated  Correlated  Anticorrelated  NS  Equivocal**  Equivocal***  NS  NS  NS | *T. crocea* [*^1^*](https://www.zotero.org/google-docs/?DmA3NR)  *T. derasa* [^2^](https://www.zotero.org/google-docs/?VjmhsE)  *T. gigas* (fossil) [^3^](https://www.zotero.org/google-docs/?PGwy74)  *T. gigas* [*^4^*](https://www.zotero.org/google-docs/?UWTOpw)  *T. gigas* [^5^](https://www.zotero.org/google-docs/?x4vuJz)  *T. gigas* (fossil) [^6^](https://www.zotero.org/google-docs/?gNZ5V5)  *T. squamosa* [^6^](https://www.zotero.org/google-docs/?u0K1Uk)  *T. squamosa* [*^7^*](https://www.zotero.org/google-docs/?Hxm3XD)  *Tridacna spp.* [*^8^*](https://www.zotero.org/google-docs/?5LO7W2)  *T. squamosa* [this study]  *T. crocea* [*^1^*](https://www.zotero.org/google-docs/?pDgCVA)  *T. derasa* [^2^](https://www.zotero.org/google-docs/?oUky22)  *T. gigas* [^5^](https://www.zotero.org/google-docs/?KTAcAt)  *T. gigas* (fossil) [^6^](https://www.zotero.org/google-docs/?m5im90)  *T. squamosa* [^6^](https://www.zotero.org/google-docs/?yjrQZl)  *T. squamosa* [*^7^*](https://www.zotero.org/google-docs/?ke2L0T)  *Tridacna spp.* [*^8^*](https://www.zotero.org/google-docs/?ohZe5u)  *T. squamosa* [this study]  *T. crocea* [*^1^*](https://www.zotero.org/google-docs/?rDrrWP)  *T. derasa* [^2^](https://www.zotero.org/google-docs/?HlftcS)  *T. gigas* [^5^](https://www.zotero.org/google-docs/?JjSpSo)  *T. gigas* (fossil) [^6^](https://www.zotero.org/google-docs/?LhFB3U)  *T. squamosa* [^6^](https://www.zotero.org/google-docs/?WdZIwd)  *Tridacna spp.* [*^8^*](https://www.zotero.org/google-docs/?uyrAIX)  *T. squamosa* [this study] |
| Irradiance | [^88^Sr]/[^40^Ca]  [^24^Mg]/[^40^Ca]  [^137^Ba]/[^40^Ca] | Daily  Daily  Daily  Daily | Anticorrelated  Anticorrelated  Anticorrelated  Anticorrelated | *T. crocea* [*^1^*](https://www.zotero.org/google-docs/?VIXWtZ)  *T. derasa* [^2^](https://www.zotero.org/google-docs/?QgZX5A)  *T. crocea* [*^1^*](https://www.zotero.org/google-docs/?NH47IC)  *T. crocea* [*^1^*](https://www.zotero.org/google-docs/?6tTfyQ) |
| Primary Productivity | [^137^Ba]/[^40^Ca] | Seasonal  Seasonal | Correlated  Correlated | *T. gigas* [^5^](https://www.zotero.org/google-docs/?8nS9Gp)  *T. squamosa* [*^7^*](https://www.zotero.org/google-docs/?CdUMEl) |
| Precipitation | [^24^Mg]/[^40^Ca]  [^137^Ba]/[^40^Ca] | Seasonal  Seasonal | NS  Equivocal† | *T. derasa* [^2^](https://www.zotero.org/google-docs/?RQmitq)  *T. squamosa* [*^7^*](https://www.zotero.org/google-docs/?304lau) |
| *p*CO_2_ | [^88^Sr]/[^40^Ca]  [^24^Mg]/[^40^Ca]  [^137^Ba]/[^40^Ca] | Monthly  Monthly  Monthly | NS  NS  Correlated | *T. squamosa* [this study]  *T. squamosa* [this study]  *T. squamosa* [this study] |

*Weakly correlated.

**Both positive and no correlations observed, dependent on population.

***Anticorrelated with temperature with a phase lag of several months.

†Correlation during certain seasons with abnormally high precipitation.

##

## Supplemental Table 2.

Pearson correlations and summary statistics associated with pre-exposure morphometric variables in the 14 month-old juvenile *Tridacna squamosa* clams. M: mean, SD: standard deviation.

|  | Pearson’s Correlations (R^2^) | | | | |  |  |  | Summary Statistics | | | |
| --- | --- | --- | --- | --- | --- | --- | --- | --- | --- | --- | --- | --- |
|  |  | | | | |  |  |  | Pre-Exposure | | Post-Exposure | |
|  | 1. | 2. | 3. | 4. | 5. |  | Treatment | N | M | SD | M | SD |
| 1. Clam Wet Mass (g) | 1.00 | 0.93 | 0.91 | 0.95 | 0.93 |  | *Ambient*  *Elevated pCO_2_*  *Elevated Temp*  *Multistressor* | 8  8  8  8 | 5.10  3.49  5.24  2.77 | 3.50  2.30  2.61  1.04 | 8.74  6.60  7.74  4.94 | 7.93  4.08  4.75  2.32 |
| 2. Shell Width (mm) |  | 1.00 | 0.95 | 0.97 | 0.97 |  | *Ambient*  *Elevated pCO_2_*  *Elevated Temp*  *Multistressor* | 8  8  8  8 | 11.89  10.66  12.70  10.24 | 2.74  1.94  2.19  1.78 | 13.89  13.45  14.22  12.36 | 4.48  2.70  2.57  2.34 |
| 3. Shell Length (mm) |  |  | 1.00 | 0.98 | 0.95 |  | *Ambient*  *Elevated pCO_2_*  *Elevated Temp*  *Multistressor* | 8  8  8  8 | 38.63  33.99  38.87  31.77 | 9.24  7.93  8.77  5.50 | 42.57  41.62  43.49  38.28 | 12.55  9.30  8.80  7.62 |
| 4. Shell Height (mm) |  |  |  | 1.00 | 0.95 |  | *Ambient*  *Elevated pCO_2_*  *Elevated Temp*  *Multistressor* | 8  8  8  8 | 20.76  18.37  21.54  17.33 | 5.32  4.12  3.84  2.48 | 24.53  23.12  24.55  21.51 | 8.12  5.36  4.67  4.32 |
| 5. Ornamentation Width (mm) |  |  |  |  | 1.00 |  | *Ambient*  *Elevated pCO_2_*  *Elevated Temp*  *Multistressor* | 8  8  8  8 | 16.52  14.15  16.68  12.60 | 4.39  4.07  4.59  2.73 | 20.48  19.21  19.93  17.20 | 8.21  5.12  6.07  5.02 |

N = 32 for Pearson’s correlations; All correlations are statistically significant (P < 0.0001).

## Supplemental Table 3.

Results of Levene’s *F-*Tests examining homogeneity of variances across the five shell morphometric variables, the three organic content variables, and nine mineralogical component variables of newly-formed scute and shell in the fluted giant clam *T. squamosa*.

| Trait | Levene’s *F* Test | |
| --- | --- | --- |
|  | F_(3, 28)_ | *P* |
| Wet Mass Gain (% change) | 1.08 | 0.37 |
| Shell Length Gain (% change) | 1.07 | 0.38 |
| Shell Width Gain (% change) | 4.20 | 0.01 * |
| Shell Height Gain (% change) | 0.74 | 0.54 |
| Ornamentation Width (% change) | 1.21 | 0.33 |
|  | F_(3, 22)_ | *p* |
| Carbon (weight %) | 2.14 | 0.12 |
| Nitrogen (weight %) | 8.15 | < 0.01 * |
| Hydrogen (weight %) | 0.29 | 0.83 |
|  | F_(3,27)_  Scute / Shell | *P*  Scute / Shell |
| [Magnesium]  (mmol kg^-1^) | 3.79 / 1.70 | 0.02 * / 0.19 |
| [Phosphorous]  (mmol kg^-1^) | 1.98 / 4.64 | 0.14 / 0.01 * |
| [Potassium]  (mmol kg^-1^) | 3.60 / 6.46 | 0.03 * / < 0.01 * |
| [Strontium]  (mmol kg^-1^) | 2.80 / 2.98 | 0.06 ˟ / 0.05 * |
| [Calcium]  (mmol kg^-1^) | F_(3, 26)_ / F_(3, 27)_  6.65 / 1.46 | < 0.01 * / 0.25 |
| [Silicon]  (mmol kg^-1^) | F_(3, 26)_ / F_(3, 26)_  1.84 / 1.72 | 0.16 / 0.19 |
| [Manganese]  (mmol kg^-1^) | 1.79 / 3.47 | 0.17 / 0.03 * |
| [Barium]  (mmol kg^-1^) | F_(3, 26)_ / F_(3, 27)_  1.25 / 4.53 | 0.31 / 0.01 * |
| [Magnesium] / [Calcium]  (mmol / mol) | F_(3, 26)_ / F_(3, 27)_  1.04 / 1.45 | 0.39 / 0.25 |
| [Strontium] / [Calcium]  (mmol / mol) | F_(3, 26)_ / F_(3, 27)_  1.98 / 0.69 | 0.14 / 0.56 |
| [Barium] / [Calcium]  (µmol /mol) | F_(3, 25)_ / F_(3, 27)_  0.86 / 1.83 | 0.47 / 0.17 |

Significant (*) or marginal (˟) *F*-test results indicating unequal variances across treatments. F: F-value, *P*: p-value. *ANCOVA* results for minerals are based on square-root transformed data.

##

## Supplemental Table 4.

Results of 2-way *ANCOVA*s examining the effect of elevated seawater temperature and *p*CO_2_ on shell morphometric and organic traits in the fluted giant clam *Tridacna squamosa*.

|  | ***ANCOVA*** | | | |  | ***Summary Statistics*** | | | |
| --- | --- | --- | --- | --- | --- | --- | --- | --- | --- |
| **Trait** | **Factor** | **F_(1, 27)_** | ***P*** | ***η_p_^2^*** |  | ***Treatment*** | ***N*** | **M** | **SD** |
| Total Wet Mass Gain  (% change) | Temperature  *p*CO_2_  Interaction  Initial Wet Mass (cov) | 0.25  6.03  0.09  3.55 | 0.62  0.02 *  0.77  0.07 ˟ | 0.01  0.18  0.00  0.12 |  | *Ambient*  *Elevated pCO_2_*  *Elevated Temp*  *Multistressor* | 8  8  8  8 | 54.43  90.36  46.43  70.80 | 43.93  40.34  23.70  33.62 |
| Shell Length Gain  (% change) | Temperature  *p*CO_2_  Interaction  Initial Length (cov) | 0.80  9.43  1.19  0.00 | 0.32  < 0.01 *  0.29  0.96 | 0.03  0.26  0.04  0.00 |  | *Ambient*  *Elevated pCO_2_*  *Elevated Temp*  *Multistressor* | 8  8  8  8 | 9.12  22.83  13.04  19.96 | 8.34  9.05  9.55  7.25 |
| Shell Width Gain  (% change) | Temperature  *p*CO_2_  Interaction  Initial Width (cov) | 0.84  8.01  0.01  3.77 | 0.37  0.01 *  0.91  0.06 ˟ | 0.03  0.23  0.00  0.12 |  | *Ambient*  *Elevated pCO_2_*  *Elevated Temp*  *Multistressor* | 8  8  8  8 | 14.96  25.94  12.04  20.49 | 13.30  9.27  5.29  7.33 |
| Shell Height Gain  (% change) | Temperature  *p*CO_2_  Interaction  Initial Height (cov) | 0.37  4.85  0.02  3.03 | 0.55  0.04 *  0.88  0.09 ˟ | 0.01  0.15  0.00  0.10 |  | *Ambient*  *Elevated pCO_2_*  *Elevated Temp*  *Multistressor* | 8  8  8  8 | 16.53  25.95  14.09  23.12 | 12.14  11.60  7.53  9.82 |
| Ornamentation Width  (% change) | Temperature  *p*CO_2_  Interaction  Initial Ornamentation Width (cov) | 0.02  5.66  0.00  1.99 | 0.90  0.02 *  0.98  0.17 | 0.00  0.17  0.00  0.07 |  | *Ambient*  *Elevated pCO_2_*  *Elevated Temp*  *Multistressor* | 8  8  8  8 | 20.52  37.02  19.70  34.74 | 19.27  16.65  11.16  15.11 |
|  |  | F_(1, 21)_ | *p* | *η_p_^2^* |  |  |  |  |  |
| Percent Carbon  (weight %) | Temperature  *p*CO_2_  Interaction  Post-exposure Wet Mass(cov) | 1.06  0.01  0.22  0.37 | 0.32  0.91  0.64  0.55 | 0.05  0.00  0.01  0.02 |  | *Ambient*  *Elevated pCO_2_*  *Elevated Temp*  *Multistressor* | 6  7  7  6 | 12.18  12.22  12.37  12.30 | 0.11  0.08  0.42  0.42 |
| Percent Nitrogen  (weight %) | Temperature  *p*CO_2_  Interaction  Initial Wet Mass (cov) | F_(1, 20)_  3.10  2.43  0.28  0.52 | 0.09 ˟  0.13  0.60  0.48 | 0.13  0.11  0.01  0.03 |  | *Ambient*  *Elevated pCO_2_*  *Elevated Temp*  *Multistressor* | 6  7  7  5 | 0.09  0.03  0.03  0.00 | 0.12  0.04  0.03  0.00 |
| Percent Hydrogen  (weight %) | Temperature  *p*CO_2_  Interaction  Initial Wet Mass (cov) | 0.01  0.38  1.61  0.03 | 0.91  0.54  0.22  0.87 | 0.00  0.01  0.07  0.00 |  | *Ambient*  *Elevated pCO_2_*  *Elevated Temp*  *Multistressor* | 6  7  7  6 | 0.14  0.15  0.15  0.13 | 0.03  0.02  0.02  0.04 |

Trait: response variable, Factor:independent variable incl. fixed factors and covariate (cov), F: F-value, *P*: p-value, *η_p_^2^:* partial eta squared, N: number of samples, M: group mean (% change), and SD: group standard deviation. Factors with significant (*) or marginal effects (˟) on investigated traits are also noted.

## Supplemental Table 5.

Results of 2-way *ANCOVA*s examining the effect of elevated seawater temperature and *p*CO_2_ on mineral concentrations of newly-formed scute in the fluted giant clam *T. squamosa*.

|  | ***ANCOVA*** | | | |  | **Summary Statistics** | | | |
| --- | --- | --- | --- | --- | --- | --- | --- | --- | --- |
| **Trait** | **Factor** | **F_(1, 26)_** | **P** | ***η_p_^2^*** |  | **Treatment** | **N** | **M** | **SD** |
| [Calcium]  (mmol kg^-1^) | *p*CO_2_  Temperature  Interaction  Post-exposure Wet Mass (cov) | F_(1, 25)_  2.40  8.05  3.04  10.67 | 0.13  0.01 *  0.09 ˟  < 0.01 * | 0.09  0.24  0.11  0.30 |  | *Ambient*  *Elevated pCO_2_*  *Elevated Temp*  *Multistressor* | 6  8  8  8 | 4700.37  8636.72  19178.69  10122.93 | 3111.39  5014.59  21800.54  9164.46 |
| [Magnesium]  (mmol kg^-1^) | *p*CO_2_  Temperature  Interaction  Post-exposure Wet Mass (cov) | 0.06  1.27  0.17  8.00 | 0.81  0.27  0.69  0.01 * | 0.00  0.05  0.01  0.24 |  | *Ambient*  *Elevated pCO_2_*  *Elevated Temp*  *Multistressor* | 7  8  8  8 | 16.28  5.76  13.81  7.84 | 35.85  2.99  14.70  6.58 |
| [Manganese]  (mmol kg^-1^) | *p*CO_2_  Temperature  Interaction  Post-exposure Wet Mass (cov) | 0.81  1.10  1.47  3.08 | 0.38  0.30  0.24  0.09 | 0.03  0.04  0.05  0.11 |  | *Ambient*  *Elevated pCO_2_*  *Elevated Temp*  *Multistressor* | 7  8  8  8 | 0.01  0.01  0.01  0.01 | 0.01  0.01  0.02  0.01 |
| [Strontium]  (mmol kg^-1^) | *p*CO_2_  Temperature  Interaction  Post-exposure Wet Mass (cov) | 0.12  1.61  0.15  8.79 | 0.73  0.22  0.70  0.01 * | 0.00  0.06  0.01  0.25 |  | *Ambient*  *Elevated pCO_2_*  *Elevated Temp*  *Multistressor* | 7  8  8  8 | 46.49  22.11  51.34  31.89 | 97.64  19.36  62.21  26.57 |
| [Barium]  (mmol kg^-1^) | *p*CO_2_  Temperature  Interaction  Post-exposure Wet Mass (cov) | F_(1, 25)_  4.20  0.10  0.01  8.26 | 0.05 *  0.77  0.92  0.01 * | 0.14  0.00  0.00  0.25 |  | *Ambient*  *Elevated pCO_2_*  *Elevated Temp*  *Multistressor* | 7  8  7  8 | 0.01  0.01  0.01  0.02 | 0.01  0.01  0.02  0.02 |
| [Silicon]  (mmol kg^-1^) | *p*CO_2_  Temperature  Interaction  Post-exposure Wet Mass (cov) | F_(1, 25)_  1.45  0.24  0.26  3.61 | 0.24  0.63  0.61  0.07 ˟ | 0.05  0.01  0.01  0.13 |  | *Ambient*  *Elevated pCO_2_*  *Elevated Temp*  *Multistressor* | 6  8  8  8 | 9.62  4.72  16.64  13.82 | 8.78  4.34  24.98  18.54 |
| [Phosphorus^-^]  (mmol kg^-1^) | *p*CO_2_  Temperature  Interaction  Post-exposure Wet Mass (cov) | 0.33  0.88  0.00  0.61 | 0.57  0.36  0.99  0.44 | 0.01  0.03  0.00  0.02 |  | *Ambient*  *Elevated pCO_2_*  *Elevated Temp*  *Multistressor* | 7  8  8  8 | 4.17  2.02  5.75  3.56 | 5.88  1.01  6.99  2.49 |
| [Potassium]  (mmol kg^-1^) | *p*CO_2_  Temperature  Interaction  Post-exposure Wet Mass (cov) | 0.00  0.25  0.05  7.27 | 0.95  0.62  0.82  0.01 * | 0.00  0.01  0.00  0.22 |  | *Ambient*  *Elevated pCO_2_*  *Elevated Temp*  *Multistressor* | 7  8  8  8 | 7.43  2.69  5.86  2.68 | 15.22  1.71  6.89  2.19 |
| [^24^Mg]/[^40^Ca]  (mmol/mol) | *p*CO_2_  Temperature  Interaction  Post-exposure Wet Mass (cov) | F_(1, 25)_  1.76  2.24  0.37  1.39 | 0.20  0.15  0.55  0.25 | 0.07  0.08  0.01  0.05 |  | *Ambient*  *Elevated pCO_2_*  *Elevated Temp*  *Multistressor* | 6  8  8  8 | 0.58  0.76  0.77  0.85 | 0.32  0.23  0.10  0.21 |
| [^88^Sr]/[^40^Ca]  (mmol/mol) | *p*CO_2_  Temperature  Interaction  Post-exposure Wet Mass (cov) | F_(1, 25)_  0.02  0.01  0.37  0.23 | 0.88  0.94  0.55  0.63 | 0.00  0.00  0.01  0.01 |  | *Ambient*  *Elevated pCO_2_*  *Elevated Temp*  *Multistressor* | 6  8  8  8 | 2.58  2.51  2.68  3.32 | 2.49  1.14  0.72  1.76 |
| [^137^Ba]/[^40^Ca]  (µmol/mol) | *p*CO_2_  Temperature  Interaction  Post-exposure Wet Mass (cov) | F_(1, 24)_  0.32  0.07  0.08  0.20 | 0.58  0.79  0.78  0.66 | 0.01  0.00  0.00  0.01 |  | *Ambient*  *Elevated pCO_2_*  *Elevated Temp*  *Multistressor* | 6  8  7  8 | 1.23  1.61  0.99  1.66 | 1.58  0.78  1.88  1.13 |

Trait: response variable, Factor: incl. fixed factors and covariate (cov), F: F-value, *P*: p-value, *η_p_^2^:* partial eta squared, N: number of samples, M: group mean (mmol kg^-1^), and SD: group standard deviation. Factors with significant (*) or marginal effects (˟) on investigated traits are also noted. *ANCOVA* results are based on square-root transformed data whereas summary statistics are for non-transformed data.

## Supplemental Table 6.

Results of 2-way *ANCOVA*s examining the effect of elevated seawater temperature and *p*CO_2_ on mineral concentrations of newly-formed shell in the fluted giant clam *T. squamosa*.

|  | ***ANCOVA*** | | | |  | **Summary Statistics** | | | |
| --- | --- | --- | --- | --- | --- | --- | --- | --- | --- |
| **Trait** | **Factor** | **F_(1, 26)_** | **P** | ***η_p_^2^*** |  | **Treatment** | **N** | **M** | **SD** |
| [Calcium]  (mmol kg^-1^) | *p*CO_2_  Temperature  Interaction  Post-exposure Wet Mass (cov) | 0.23  0.10  1.45  0.23 | 0.64  0.76  0.24  0.63 | 0.01  0.00  0.05  0.01 |  | *Ambient*  *Elevated pCO_2_*  *Elevated Temp*  *Multistressor* | 8  8  8  7 | 17896.24  27884.13  18606.54  56968.57 | 20342.80  50050.16  13970.41  38836.03 |
| [Magnesium]  (mmol kg^-1^) | *p*CO_2_  Temperature  Interaction  Post-exposure Wet Mass (cov) | 0.30  0.40  1.36  0.03 | 0.59  0.53  0.86  0.25 | 0.01  0.02  0.05  0.00 |  | *Ambient*  *Elevated pCO_2_*  *Elevated Temp*  *Multistressor* | 8  8  8  7 | 9.99  15.72  13.04  40.26 | 13.32  26.88  11.25  28.10 |
| [Manganese]  (mmol kg^-1^) | *p*CO_2_  Temperature  Interaction  Post-exposure Wet Mass (cov) | 0.37  0.07  0.66  0.70 | 0.55  0.79  0.42  0.41 | 0.01  0.00  0.02  0.03 |  | *Ambient*  *Elevated pCO_2_*  *Elevated Temp*  *Multistressor* | 8  8  8  7 | 0.00  0.01  0.01  0.02 | 0.00  0.02  0.00  0.02 |
| [Strontium]  (mmol kg^-1^) | *p*CO_2_  Temperature  Interaction  Post-exposure Wet Mass (cov) | 1.03  0.41  0.10  0.25 | 0.32  0.53  0.75  0.62 | 0.04  0.02  0.00  0.01 |  | *Ambient*  *Elevated pCO_2_*  *Elevated Temp*  *Multistressor* | 8  8  8  7 | 30.99  120.86  50.07  132.97 | 25.03  275.85  40.67  114.85 |
| [Barium]  (mmol kg^-1^) | *p*CO_2_  Temperature  Interaction  Post-exposure Wet Mass (cov) | 4.62  0.10  0.75  0.99 | 0.04 *  0.76  0.39  0.33 | 0.15  0.00  0.03  0.04 |  | *Ambient*  *Elevated pCO_2_*  *Elevated Temp*  *Multistressor* | 8  8  8  7 | 0.00  0.03  0.01  0.06 | 0.01  0.05  0.01  0.05 |
| [Silicon]  (mmol kg^-1^) | *p*CO_2_  Temperature  Interaction  Post-exposure Wet Mass (cov) | F_(1, 25)_  1.04  0.00  0.16  3.70 | 0.32  1.00  0.69  0.07 ˟ | 0.04  0.00  0.01  0.13 |  | *Ambient*  *Elevated pCO_2_*  *Elevated Temp*  *Multistressor* | 8  8  8  6 | 24.04  17.07  40.43  27.52 | 29.99  35.85  77.35  37.41 |
| [Phosphorus^-^]  (mmol kg^-1^) | *p*CO_2_  Temperature  Interaction  Post-exposure Wet Mass (cov) | 0.08  0.34  0.73  2.55 | 0.78  0.56  0.40  0.12 | 0.00  0.01  0.03  0.09 |  | *Ambient*  *Elevated pCO_2_*  *Elevated Temp*  *Multistressor* | 8  8  8  7 | 3.30  3.36  7.81  14.52 | 3.34  3.75  11.89  19.17 |
| [Potassium]  (mmol kg^-1^) | *p*CO_2_  Temperature  Interaction  Post-exposure Wet Mass (cov) | 0.03  0.77  3.79  0.02 | 0.86  0.39  0.06 ˟  0.90 | 0.00  0.02  0.13  0.00 |  | *Ambient*  *Elevated pCO_2_*  *Elevated Temp*  *Multistressor* | 8  8  8  7 | 4.35  4.53  1.78  11.69 | 3.73  7.51  0.80  11.15 |
| [^24^Mg]/[^40^Ca]  (mmol/mol) | *p*CO_2_  Temperature  Interaction  Post-exposure Wet Mass (cov) | 2.65  7.38  1.94  3.23 | 0.12  0.01 *  0.17  0.08 ˟ | 0.09  0.22  0.07  0.11 |  | *Ambient*  *Elevated pCO_2_*  *Elevated Temp*  *Multistressor* | 8  8  8  7 | 0.49  0.60  0.65  0.66 | 0.14  0.09  0.08  0.14 |
| [^88^Sr]/[^40^Ca]  (mmol/mol) | *p*CO_2_  Temperature  Interaction  Post-exposure Wet Mass (cov) | 0.31  0.54  0.87  0.00 | 0.58  0.47  0.36  0.99 | 0.01  0.02  0.03  0.00 |  | *Ambient*  *Elevated pCO_2_*  *Elevated Temp*  *Multistressor* | 8  8  8  7 | 2.30  2.70  2.81  2.28 | 1.97  1.29  1.00  0.76 |
| [^137^Ba]/[^40^Ca]  (µmol/mol) | *p*CO_2_  Temperature  Interaction  Post-exposure Wet Mass (cov) | 8.98  0.92  2.01  0.28 | < 0.01 *  0.35  0.17  0.60 | 0.26  0.03  0.07  0.01 |  | *Ambient*  *Elevated pCO_2_*  *Elevated Temp*  *Multistressor* | 8  8  8  7 | 0.27  1.17  0.55  0.82 | 0.44  0.46  0.76  0.71 |

Trait: response variable, Factor: incl. fixed factors and covariate (cov), F: F-value, *P*: p-value, *η_p_^2^:* partial eta squared, N: number of samples, M: group mean (mmol kg^-1^), and SD: group standard deviation. Factors with significant (*) or marginal effects (˟) on investigated traits are also noted. *ANCOVA* results are based on square-root transformed data whereas summary statistics are for non-transformed data.

## Supplemental Table 7.

Results of 2-way *ANCOVA*s examining the effect of elevated seawater temperature and *p*CO_2_ on mineral concentrations of older-growth scute in the fluted giant clam *T. squamosa*.

|  | ***ANCOVA*** | | | |  | **Summary Statistics** | | | |
| --- | --- | --- | --- | --- | --- | --- | --- | --- | --- |
| **Trait** | **Factor** | **F_(1, 23)_** | **P** | ***η_p_^2^*** |  | **Treatment** | **N** | **M** | **SD** |
| [Calcium]  (mmol kg^-1^) | *p*CO_2_  Temperature  Interaction  Post-exposure Wet Mass (cov) | 1.87 0.42 0.30 3.22 | 0.18 0.52 0.59  0.09 ˟ | 0.08 0.02 0.01 0.12 |  | *Ambient*  *Elevated pCO_2_*  *Elevated Temp*  *Multistressor* | 7  8  7  6 | 3148.89  8228.72  4147.37  6508.34 | 3854.65  9604.53  3854.88  10739.02 |
| [Magnesium]  (mmol kg^-1^) | *p*CO_2_  Temperature  Interaction  Post-exposure Wet Mass (cov) | 1.96  1.17  1.33  1.79 | 0.17  0.29  0.26  0.19 | 0.08  0.05  0.05  0.07 |  | *Ambient*  *Elevated pCO_2_*  *Elevated Temp*  *Multistressor* | 7  8  7  6 | 3.70  9.85  7.48  6.22 | 4.55  11.00  8.01  11.27 |
| [Manganese]  (mmol kg^-1^) | *p*CO_2_  Temperature  Interaction  Post-exposure Wet Mass (cov) | 2.04  0.32 0.89 0.91 | 0.17  0.58  0.36  0.35 | 0.08  0.01  0.04  0.04 |  | *Ambient*  *Elevated pCO_2_*  *Elevated Temp*  *Multistressor* | 7  8  7  6 | 0.01  0.03  0.01  0.01 | 0.01  0.03  0.01  0.02 |
| [Strontium]  (mmol kg^-1^) | *p*CO_2_  Temperature  Interaction  Post-exposure Wet Mass (cov) | 3.65 1.39 0.43 3.84 | 0.07 ˟ 0.25 0.52 0.06 ˟ | 0.14  0.06  0.02  0.14 |  | *Ambient*  *Elevated pCO_2_*  *Elevated Temp*  *Multistressor* | 7  8  7  6 | 5.29  26.21  12.63  26.86 | 8.59  38.68  10.24  39.24 |
| [Barium]  (mmol kg^-1^) | *p*CO_2_  Temperature  Interaction  Post-exposure Wet Mass (cov) | F_(1, 24)_  5.00 0.26 0.05 9.08 | 0.03 * 0.61 0.83  0.01 * | 0.17 0.01  0.00  0.27 |  | *Ambient*  *Elevated pCO_2_*  *Elevated Temp*  *Multistressor* | 7  8  7  7 | 0.01  0.02  0.01  0.03 | 0.01  0.03  0.01  0.04 |
| [Silicon]  (mmol kg^-1^) | *p*CO_2_  Temperature  Interaction  Post-exposure Wet Mass (cov) | F_(1, 24)_  0.84 0.19 0.01 0.05 | 0.37 0.67 0.92 0.82 | 0.03 0.01 0.00 0.00 |  | *Ambient*  *Elevated pCO_2_*  *Elevated Temp*  *Multistressor* | 7  8  7  7 | 6.17  32.31  15.71  39.18 | 3.10  57.67  21.21  57.28 |
| [Phosphorus^-^]  (mmol kg^-1^) | *p*CO_2_  Temperature  Interaction  Post-exposure Wet Mass (cov) | 2.86 1.82 0.62 0.38 | 0.10 0.19 0.44 0.54 | 0.11  0.07  0.03  0.02 |  | *Ambient*  *Elevated pCO_2_*  *Elevated Temp*  *Multistressor* | 7  8  7  6 | 1.94 9.12 7.51 9.92 | 1.37 9.80 9.04 13.08 |
| [Potassium]  (mmol kg^-1^) | *p*CO_2_  Temperature  Interaction  Post-exposure Wet Mass (cov) | 2.80  1.39  1.41 2.46 | 0.11 0.25 0.25 0.13 | 0.11  0.06  0.06  0.10 |  | *Ambient*  *Elevated pCO_2_*  *Elevated Temp*  *Multistressor* | 7  8  7  6 | 1.62 6.01 4.35 3.48 | 2/05 6.48  5.61  4.04 |
| [^24^Mg]/[^40^Ca]  (mmol/mol) | *p*CO_2_  Temperature  Interaction  Post-exposure Wet Mass (cov) | F_(1, 23)_  1.00 1.65 0.30 0.62 | 0.33 0.21  0.59  0.44 | 0.04  0.07  0.01  0.03 |  | *Ambient*  *Elevated pCO_2_*  *Elevated Temp*  *Multistressor* | 7  8  7  6 | 8.78 3.95 1.59 1.52 | 20.69 7.00 0.59  1.25 |
| [^88^Sr]/[^40^Ca]  (mmol/mol) | *p*CO_2_  Temperature  Interaction  Post-exposure Wet Mass (cov) | F_(1, 23)_  1.32  0.00  0.67 0.49 | 0.26  0.95  0.42  0.49 | 0.05 0.00 0.03  0.02 |  | *Ambient*  *Elevated pCO_2_*  *Elevated Temp*  *Multistressor* | 7  8  7  6 | 2.65 7.51 3.17 3.77 | 2.58 12.27  1.28  2.32 |
| [^137^Ba]/[^40^Ca]  (µmol/mol) | *p*CO_2_  Temperature  Interaction  Post-exposure Wet Mass (cov) | F_(1, 22)_  0.98 0.01 0.60 0.19 | 0.33 0.94 0.45  0.67 | 0.04 0.00 0.03 0.01 |  | *Ambient*  *Elevated pCO_2_*  *Elevated Temp*  *Multistressor* | 7  8  7  6 | 1.83 3.74 1.89 1.40 | 2.39  6.40  2.92  1.33 |

Trait: response variable, Factor: incl. fixed factors and covariate (cov), F: F-value, *P*: p-value, *η_p_^2^:* partial eta squared, N: number of samples, M: group mean (mmol kg^-1^), and SD: group standard deviation. Factors with significant (*) or marginal effects (˟) on investigated traits are also noted. *ANCOVA* results are based on square-root transformed data whereas summary statistics are for non-transformed data.

## Supplemental Table 8.

Results of 2-way *ANCOVA*s examining the effect of elevated temperature and *p*CO_2_ on mineral concentrations of older-growth shell in the fluted giant clam *T. squamosa*.

|  | ***ANCOVA*** | | | |  | **Summary Statistics** | | | |
| --- | --- | --- | --- | --- | --- | --- | --- | --- | --- |
| **Trait** | **Factor** | **F_(1, 26)_** | **P** | ***η_p_^2^*** |  | **Treatment** | **N** | **M** | **SD** |
| [Calcium]  (mmol kg^-1^) | *p*CO_2_  Temperature  Interaction  Post-exposure Wet Mass (cov) | 1.37 3.91 4.67 0.33 | 0.25 0.06 ˟ 0.04 * 0.57 | 0.05 0.13 0.15 0.01 |  | *Ambient*  *Elevated pCO_2_*  *Elevated Temp*  *Multistressor* | 7  8  8  8 | 109510.05  35689.52  12553.36  80249.66 | 156705.07  40808.94  6801.87  126975.81 |
| [Magnesium]  (mmol kg^-1^) | *p*CO_2_  Temperature  Interaction  Post-exposure Wet Mass (cov) | 2.69 6.18  5.72  0.01 | 0.11 0.02 * 0.02 *  0.93 | 0.09 0.19 0.18 0.00 |  | *Ambient*  *Elevated pCO_2_*  *Elevated Temp*  *Multistressor* | 7  8  8  8 | 84.46 19.89 6.55 48.12 | 116.20  17.32  3.26  71.96 |
| [Manganese]  (mmol kg^-1^) | *p*CO_2_  Temperature  Interaction  Post-exposure Wet Mass (cov) | 4.61 7.75 6.98 0.60 | 0.04 * 0.01 * 0.01 *  0.45 | 0.15 0.23 0.21 0.02 |  | *Ambient*  *Elevated pCO_2_*  *Elevated Temp*  *Multistressor* | 7  8  8  8 | 0.17  0.02  0.00  0.08 | 0.25  0.02  0.00  0.10 |
| [Strontium]  (mmol kg^-1^) | *p*CO_2_  Temperature  Interaction  Post-exposure Wet Mass (cov) | 0.00 1.28 2.01 1.29 | 0.99  0.27  0.17  0.27 | 0.00 0.05 0.07  0.05 |  | *Ambient*  *Elevated pCO_2_*  *Elevated Temp*  *Multistressor* | 7  8  8  8 | 153.81  140.72 33.53  203.52 | 212.22 237.19 21.82 21.03 |
| [Barium]  (mmol kg^-1^) | *p*CO_2_  Temperature  Interaction  Post-exposure Wet Mass (cov) | 0.82 3.12 3.70 0.57 | 0.37 0.09 0.07 ˟ 0.46 | 0.03 0.11 0.12 0.02 |  | *Ambient*  *Elevated pCO_2_*  *Elevated Temp*  *Multistressor* | 7  8  8  8 | 0.25 0.09 0.03 0.23 | 0.36 0.13 0.01 0.44 |
| [Silicon]  (mmol kg^-1^) | *p*CO_2_  Temperature  Interaction  Post-exposure Wet Mass (cov) | 2.80 6.23 4.81  0.54 | 0.11  0.02 * 0.04 * 0.47 | 0.10 0.19 0.16 0.02 |  | *Ambient*  *Elevated pCO_2_*  *Elevated Temp*  *Multistressor* | 7  8  8  8 | 58.14 15.02 5.26 28.72 | 77.77 14.48 5.24 31.21 |
| [Phosphorus^-^]  (mmol kg^-1^) | *p*CO_2_  Temperature  Interaction  Post-exposure Wet Mass (cov) | 1.70 5.54 5.10 0.02 | 0.20 0.03 * 0.03 * 0.88 | 0.06 0.18 0.16 0.00 |  | *Ambient*  *Elevated pCO_2_*  *Elevated Temp*  *Multistressor* | 7  8  8  8 | 26.49 9.75 2.45  17.98 | 33.86 11.28 0.96 24.02 |
| [Potassium]  (mmol kg^-1^) | *p*CO_2_  Temperature  Interaction  Post-exposure Wet Mass (cov) | 2.10 6.22 5.27  0.01 | 0.16  0.02 *  0.03 *  0.92 | 0.07 0.19 0.17 0.00 |  | *Ambient*  *Elevated pCO_2_*  *Elevated Temp*  *Multistressor* | 7  8  8  8 | 41.43 12.41 2.85 24.09 | 55.33 13.34 0.79 34.91 |
| [^24^Mg]/[^40^Ca]  (mmol/mol) | *p*CO_2_  Temperature  Interaction  Post-exposure Wet Mass (cov) | 1.30 5.16 0.05 8.66 | 0.26 0.03 * 0.83  0.01 * | 0.05 0.16 0.00 0.24 |  | *Ambient*  *Elevated pCO_2_*  *Elevated Temp*  *Multistressor* | 7  8  8  8 | 0.82 0.75 0.59 0.56 | 0.27 0.30 0.25 0.23 |
| [^88^Sr]/[^40^Ca]  (mmol/mol) | *p*CO_2_  Temperature  Interaction  Post-exposure Wet Mass (cov) | 0.55 0.37 0.20 0.60 | 0.47 0.55 0.66 0.44 | 0.02 0.01 0.01 0.02 |  | *Ambient*  *Elevated pCO_2_*  *Elevated Temp*  *Multistressor* | 7  8  8  8 | 1.73 2.97 2.90 2.87 | 0.53 1.45 1.42 1.81 |
| [^137^Ba]/[^40^Ca]  (µmol/mol) | *p*CO_2_  Temperature  Interaction  Post-exposure Wet Mass (cov) | 0.33  0.01 0.24 2.15 | 0.57 0.91 0.63 0.15 | 0.01  0.00  0.01  0.08 |  | *Ambient*  *Elevated pCO_2_*  *Elevated Temp*  *Multistressor* | 7  8  8  8 | 2.38 2.51 2.35 2.17 | 0.42  0.98  1.02  0.95 |

Trait: response variable, Factor: incl. fixed factors and covariate (cov), F: F-value, *P*: p-value, *η_p_^2^:* partial eta squared, N: number of samples, M: group mean (mmol kg^-1^), and SD: group standard deviation. Factors with significant (*) or marginal effects (˟) on investigated traits are also noted. *ANCOVA* results are based on square-root transformed data whereas summary statistics are for non-transformed data.

## References

[1. Warter, V., Erez, J. & Müller, W. Environmental and physiological controls on daily trace element incorporation in *Tridacna crocea* from combined laboratory culturing and ultra-high resolution LA-ICP-MS analysis. *Palaeogeogr. Palaeoclimatol. Palaeoecol.* **496**, 32–47 (2018).](https://www.zotero.org/google-docs/?YZ6CDe)

[2. Sano, Y. *et al.* Past daily light cycle recorded in the strontium/calcium ratios of giant clam shells. *Nat. Commun.* **3**, 761 (2012).](https://www.zotero.org/google-docs/?YZ6CDe)

[3. Yan, H., Shao, D., Wang, Y. & Sun, L. Sr/Ca profile of long-lived *Tridacna gigas* bivalves from South China Sea: A new high-resolution SST proxy. *Geochim. Cosmochim. Acta* **112**, 52–65 (2013).](https://www.zotero.org/google-docs/?YZ6CDe)

[4. Yan, H., Sun, L., Shao, D. & Wang, Y. Seawater temperature seasonality in the South China Sea during the late Holocene derived from high-resolution Sr/Ca ratios of *Tridacna gigas*. *Quat. Res.* **83**, 298–306 (2015).](https://www.zotero.org/google-docs/?YZ6CDe)

[5. Elliot, M. *et al.* Profiles of trace elements and stable isotopes derived from giant long-lived *Tridacna gigas* bivalves: Potential applications in paleoclimate studies. *Palaeogeogr. Palaeoclimatol. Palaeoecol.* **280**, 132–142 (2009).](https://www.zotero.org/google-docs/?YZ6CDe)

[6. Batenburg, S. J. *et al.* Interannual climate variability in the Miocene: High resolution trace element and stable isotope ratios in giant clams. *Palaeogeogr. Palaeoclimatol. Palaeoecol.* **306**, 75–81 (2011).](https://www.zotero.org/google-docs/?YZ6CDe)

[7. Arias-Ruiz, C. *et al.* Geochemical fingerprints of climate variation and the extreme La Niña 2010–11 as recorded in a *Tridacna squamosa* shell from Sulawesi, Indonesia. *Palaeogeogr. Palaeoclimatol. Palaeoecol.* **487**, 216–228 (2017).](https://www.zotero.org/google-docs/?YZ6CDe)

[8. Warter, V., Mueller, W., Wesselingh, F. P., Todd, J. A. & Renema, W. Late Miocene seasonal to subdecadal climate variability in the Indo-West Pacific (East Kalimantan, Indonesia) preserved in giant clams. *Palaios* **30**, 66–82 (2015).](https://www.zotero.org/google-docs/?YZ6CDe)
